# Association of Primary Care Physicians’ Individual- and Community-Level Characteristics With Contraceptive Service Provision to Medicaid Beneficiaries

**DOI:** 10.1001/jamahealthforum.2023.0106

**Published:** 2023-03-17

**Authors:** Mandar Bodas, Julia Strasser, Qian Luo, Ellen Schenk, Candice Chen

**Affiliations:** 1 Fitzhugh Mullan Institute for Health Workforce Equity, Department of Health Policy and Management, Milken Institute School of Public Health, The George Washington University, Washington, DC

## Abstract

**Importance:**

Little is known about primary care physicians who provide contraceptive services to Medicaid beneficiaries. Evaluating this workforce may help explain barriers to accessing these services since contraceptive care access is critical for Medicaid beneficiaries’ health.

**Objective:**

To describe the primary care physician workforce that provides contraceptive services to Medicaid beneficiaries and explore the factors associated with their Medicaid contraceptive service provision.

**Design, Setting, and Participants:**

This cross-sectional study, conducted from August 1 to October 10, 2022, used data from the Transformed Medicaid Statistical Information System from 2016 for primary care physicians from 4 specialties (family medicine, internal medicine, obstetrics and gynecology [OBGYN], and pediatrics).

**Main Outcomes and Measures:**

The main outcomes were providing intrauterine devices (IUDs) or contraceptive implants to at least 1 Medicaid beneficiary, prescribing hormonal birth control methods (including a pill, patch, or ring) to at least 1 Medicaid beneficiary, the total number of Medicaid beneficiaries provided IUDs or implants, and the total number Medicaid beneficiaries prescribed hormonal birth control methods in 2016. Physician- and community-level factors associated with contraceptive care provision were assessed using multivariate regression methods.

**Results:**

In the sample of 251 017 physicians (54% male; mean [SD] age, 49.17 [12.58] years), 28% were international medical graduates (IMGs) and 70% practiced in a state that had expanded Medicaid in 2016. Of the total physicians, 48% prescribed hormonal birth control methods while 10% provided IUDs or implants. For OBGYN physicians, compared with physicians younger than 35 years, being aged 35 to 44 years (odds ratio [OR], 3.51; 95% CI, 2.93-4.21), 45 to 54 years (OR, 3.01; 95% CI, 2.43-3.72), or 55 to 64 years (OR, 2.27; 95% CI, 1.82-2.83) was associated with higher odds of providing IUDs and implants. However, among family medicine physicians, age groups associated with lower odds of providing IUDs or implants were 45 to 54 years (OR, 0.66; 95% CI, 0.55-0.80), 55 to 64 years (OR, 0.51; 95% CI, 0.39-0.65), and 65 years or older (OR, 0.29; 95% CI, 0.19-0.44). Except for those specializing in OBGYN, being an IMG was associated with lower odds of providing hormonal contraceptive service (family medicine IMGs: OR, 0.80 [95% CI, 0.73-0.88]; internal medicine IMGs: OR, 0.85 [95% CI, 0.77-0.93]; and pediatric IMGs: OR, 0.85 [95% CI, 0.78-0.93]). Practicing in a state that expanded Medicaid by 2016 was associated with higher odds of prescribing hormonal contraception for family medicine (OR 1.50; 95% CI, 1.06-2.12) and internal medicine (OR, 1.71; 95% CI, 1.18-2.48) physicians but not for physicians from other specialties.

**Conclusions and Relevance:**

In this cross-sectional study of primary care physicians, physician- and community-level factors, such as specialty, age, and the Medicaid expansion status of their state, were significantly associated with how they provided contraceptive services to Medicaid beneficiaries. However, the existence of associations varied across clinical specialties. Ensuring access to contraception among Medicaid beneficiaries may therefore require policy and program approaches tailored for different physician types.

## Key Points

**Question:**

What factors are associated with primary care physicians providing contraceptive services to Medicaid beneficiaries?

**Findings:**

In this cross-sectional study of 251 017 primary care physicians, physician factors, such as specialty, age, and the Medicaid expansion status of their state, were significantly associated with how they provided contraceptive services to Medicaid beneficiaries.

**Meaning:**

In this study, several physician- and community-level factors were associated with Medicaid contraceptive service provision by primary care physicians; however, the findings were dissimilar across specialties and contraception methods.

## Teaser

This cross-sectional study examines the factors associated with primary care physicians providing contraceptive services to Medicaid beneficiaries.

## Introduction

Contraceptive care is a critical component of comprehensive health care that helps individuals achieve their preferred family size and birth spacing and is associated with improved health outcomes.^1^ However, this care is not uniformly accessible across the US. Disparities exist for vulnerable populations due to barriers such as lack of insurance coverage and scarcity of practitioners offering contraception care.^2,3^ Medicaid, which insures more than 87 million individuals, mostly populations with disabilities and low income, is one of the largest payers of contraception care.^4^ While it is known that the income, insurance coverage, and socioeconomic status of an individual are associated with contraceptive use,^5^ little is known about the factors associated with physicians choosing to provide contraception care, especially to Medicaid beneficiaries.

Medicaid beneficiaries often struggle to find physicians willing to see them.^6^ Nationally, approximately one-third of primary care physicians do not accept Medicaid.^7^ Medicaid beneficiaries’ wait times for appointments are longer than those of private insurance beneficiaries.^8,9^ These workforce barriers further interact with other impediments in contraception access. First, there is little consistency across states in terms of which type of contraceptive care is covered under Medicaid.^10^ While family planning services are a mandatory benefit through Medicaid, there are few specifications on what services should be covered under them. Second, contraception access differs due to the variation in Medicaid eligibility requirements.^11^ In Medicaid expansion states, a larger group of beneficiaries has coverage than in nonexpansion states. Some states additionally expand eligibility for family planning services through Section 1115 Medicaid waivers or state plan amendments. These family planning expansions increase the beneficiary pool as well as types of contraceptive services available to beneficiaries. Thus, workforce, structural, and policy factors may either hinder or facilitate Medicaid beneficiaries’ access to contraception care.

As a previous study by some of us found,^12^ clinicians from a variety of specialties and professions provide multiple types of contraception care in the US, and the rates of Medicaid acceptance differ both by clinician specialty and by state. However, other studies^13,14^ on the contraception workforce relied on surveys or other self-reported data, which could potentially introduce reporting errors. Additionally, past studies have focused on a limited sample or subset of clinicians (eg, those practicing in a single state or from a single specialty).^15^ As a result, to date, how primary care physicians provide contraceptive care to Medicaid populations is not yet known. Furthermore, it is also not clear whether and how individual-level physician characteristics and community-level factors are associated with Medicaid contraceptive care participation.

The objective of this study was to describe the primary care physician workforce that provides contraceptive services to Medicaid beneficiaries. Using multiple data sources, we aimed to comprehensively analyze this workforce and explore physician- and community-level factors associated with Medicaid contraceptive service participation.

## Methods

This cross-sectional study was conducted from August 1 to October 10, 2022. We followed the Strengthening the Reporting of Observational Studies in Epidemiology (STROBE) reporting guideline. This study was approved by The George Washington University institutional review board. Because this is a study of secondary data, it did not need informed consent. We did not collect data from participants but used previously collected data from medical claims and other sources.

### Data

The main data sources for this study were the 2016 Transformed Medicaid Statistical Information System (T-MSIS) Other Services, Pharmacy, and Annual Provider files (release 2).^16^ We identified contraceptive service provision among physicians using a modified set of *Current Procedural Terminology* codes and National Drug Codes released by the Office of Population Affairs.^17^ We used National Provider Identifiers in T-MSIS to merge T-MSIS claims data with the National Plan and Provider Enumeration System data set (January 2017).^18^ This enabled us to identify the provider type (an individual or an organization) of all active physicians. We obtained information on physicians’ sex, age, specialty, and type of medical degree they received from the American Medical Association Masterfile data set.^19^ We used data on physicians’ county-level socioeconomic factors from the Area Health Resources Files^20^ and the US Census Bureau.^21^ Data from the Kaiser Family Foundation were used to determine the Medicaid expansion status of individual states in 2016.^22^

### Sample

We limited the sample to Medicaid-participating physicians practicing in primary care specialties, including family medicine, general internal medicine, general pediatrics, and obstetrics and gynecology (OBGYN). We restricted the analysis to physicians from states that did not have any data quality issues in the 2016 T-MSIS. We excluded data from 6 states (Arkansas, Florida, Maine, Minnesota, Pennsylvania, and Rhode Island) and Washington, DC, and included data from the remaining 44 states and Puerto Rico (eTable 6 in Supplement 1). We excluded physicians for whom data on their demographic characteristics were missing. To account for the differences in patient panels of physicians practicing in different specialties, we excluded physicians who did not see any reproductive-age (15-44 years) female Medicaid beneficiaries in 2016. The analytic sample included 251 107 physicians who saw at least 1 Medicaid beneficiary in 2016 and did not have data missing on any study measure (eFigure 1 in Supplement 1).

### Measures

We included outcome measures for 2 sets of contraceptive services: (1) intrauterine devices (IUDs) or implants and (2) hormonal birth control methods, including a pill, patch, or ring (hereafter referred to as hormonal contraception). Overall, we used 4 physician-level outcome measures: (1) whether a physician provided IUDs or implants to at least 1 Medicaid beneficiary in 2016, (2) whether a physician prescribed hormonal contraception to at least 1 Medicaid beneficiary, (3) the total number of beneficiaries provided IUDs or implants, and (4) the total number of beneficiaries prescribed hormonal contraception. eTable 1 in Supplement 1 gives a list of codes used to identify contraceptive services. In multivariate analysis, we controlled for several physician-level characteristics, including their age, sex, and type of medical degree received and whether they were an international medical graduate (IMG). We assigned a state to each physician in the sample based on the number of Medicaid claims submitted. For example, if a physician was located in state A according to the National Plan and Provider Enumeration System but submitted most of their claims to the Medicaid program of state B, for the purpose of this analysis, we assigned state B as that physician’s state. A total of 18 978 physicians in the sample were reassigned states based on the number of Medicaid claims submitted (eFigures 4 and 5 in Supplement 1). Similarly, we assigned a county to each physician based on the highest number of claims from a county. Next, we used the socioeconomic characteristics of the physician’s county, including the percentage of the population with low income, and whether the county was rural (based on the Rural-Urban Continuum Codes classification scheme^23^). As an indirect measure of the demand for contraceptive services, we controlled for the population of women between ages 15 and 44 years in a physician’s county. We also used a county-level measures for the populations that belonged to certain racial and ethnic groups since these can potentially influence contraceptive choice and provision. Race and ethnicity categories included American Indian or Alaska Native, Asian, Black, Hispanic and White; details were previously reported.^24^ Finally, we controlled for the Medicaid expansion status of the physician’s state and whether the state had a Medicaid family planning waiver.

### Statistical Analysis

We began with descriptive analysis of the sample. Next, we used multivariate logistic regression models to evaluate the first and second outcomes (providing each type of contraception method to at least 1 Medicaid beneficiary) and multivariate negative binomial regression models to assess the third and fourth outcomes (the total number of beneficiaries provided each type of contraceptive method), controlling for physician- and county-level characteristics. Since it is known from past analyses^12^ that contraceptive provision patterns differ substantially among specialties, we analyzed physicians from each specialty separately. Adjusted odds ratios (ORs) for logistic regressions and average marginal effects (AMEs) for negative binomial regressions, along with 95% CIs for coefficients from both models, were calculated. Standard errors were clustered at the state level to account for heteroskedasticity. A 2-sided significance threshold of *P* = .025 was used. Stata MP, version 17 (StataCorp LLC) was used to conduct all analyses.^25^

## Results

### Descriptive Results

Table 1 describes demographic and other characteristics of the study sample. Among the sample of 251 017 physicians, the mean (SD) age was 49.17 (12.58) years; 46% were female, and 54% were male. A total of 9% graduated from schools of osteopathic medicine, 28% were IMGs, 6% practiced in rural areas, and 70% practiced in a state that had expanded Medicaid in 2016. About one-third (34%) of the sample were family medicine physicians, 34% were internal medicine physicians, 14% were OBGYN physicians, and 17% were pediatricians. The number of physicians prescribing hormonal contraceptives (121 167 [48%]) was approximately 5 times the number of physicians providing IUDs or implants (25 115 [10%]) (eTables 2 and 3 in Supplement 1). Obstetrics and gynecology physicians accounted for nearly two-thirds (16 481 of 25 115 [66%]) of the overall IUD- or implant-providing physicians, and family medicine physicians were the next-largest group of physicians (7262 [29%]) in this category (eTable 2 in Supplement 1). For each type of contraception service, family medicine and OBGYN physicians were the 2 main specialties with substantially higher mean numbers of beneficiaries treated.

**Table 1.** Demographic Characteristics of Physicians in the Study Sample^a^

Characteristic	Physicians				
	All (N = 251 017)	Family medicine (n = 86 106)	Internal medicine (n = 85 095)	OBGYN (n = 35 994)	Pediatrics (n = 43 822)
**Practice characteristics, mean (SD), No.**					
Beneficiaries provided pill, patch, or ring	5.83 (22.30)	4.42 (18.04)	1.07 (5.53)	24.63 (46.15)	2.42 (7.11)
Beneficiaries provided IUDs or implants	1.57 (19.70)	0.83 (18.63)	0.06 (4.90)	8.68 (41.87)	0.11 (2.42)
Female Medicaid beneficiaries of reproductive age seen in 2016^b^	97.59 (229.42)	101.08 (213.25)	51.11 (133.61)	249.35 (415.12)	56.37 (105.38)
**Physician characteristics, No. (%)**					
Sex					
Female	116 374 (46)	34 743 (40)	32 910 (39)	21 027 (58)	27 694 (63)
Male	134 643 (54)	51 363 (60)	52 185 (61)	14 967 (42)	16 128 (37)
Doctors of osteopathy	22 886 (9)	13 689 (16)	4792 (6)	2364 (7)	2041 (5)
International medical graduates	70 589 (28)	20 448 (24)	33 935 (40)	5190 (14)	11 016 (25)
Age, mean (SD), y	49.17 (12.58)	49.75 (12.64)	48.65 (12.40)	48.71 (12.71)	49.42 (12.63)
Age, y					
25-34	36 554 (15)	11 678 (14)	12 924 (15)	5835 (16)	6117 (14)
35-44	60 446 (24)	20 209 (23)	20 995 (25)	8730 (24)	10 512 (24)
45-54	65 942 (26)	22 125 (26)	22 927 (27)	9238 (26)	11 652 (27)
55-64	56 435 (22)	20 542 (24)	18 618 (22)	7658 (21)	9617 (22)
≥65	31 640 (13)	11 552 (13)	9631 (11)	4533 (13)	5924 (14)
Located in a rural county	15 463 (6)	9388 (11)	3365 (4)	1370 (4)	1340 (3)
**County characteristics, mean (SD)** ^c^					
County female population aged 15-44 y, No.	278 929.41 (446 138.09)	233 379.08 (427 807.56)	304 965.88 (459 168.34)	304 593.75 (451 487.91)	296 793.03 (444 326.56)
County % population below poverty line	14.43 (5.10)	14.49 (5.05)	14.49 (5.11)	14.47 (5.07)	14.17 (5.21)
Race and ethnicity					
County % population AIAN	1.58 (3.74)	1.80 (4.50)	1.45 (3.22)	1.46 (3.15)	1.48 (3.44)
County % population Asian	6.60 (7.23)	5.39 (6.40)	7.29 (7.67)	7.10 (7.46)	7.23 (7.40)
County % population Black	14.80 (13.96)	12.24 (12.99)	16.13 (14.32)	16.35 (14.30)	15.97 (14.11)
County % population Hispanic	18.05 (16.24)	16.94 (16.64)	18.24 (15.60)	18.90 (16.25)	19.15 (16.51)
County % population White	74.24 (15.50)	77.84 (14.81)	72.30 (15.64)	72.32 (15.43)	72.51 (15.35)
**State policy characteristics, No. (%)**					
Practice in a state that expanded Medicaid by 2016	174 529 (70)	56 915 (66)	62 772 (74)	24 609 (68)	30 233 (69)
Practice in a state with Medicaid family planning waiver in 2016	165 096 (66)	56 487 (66)	56 304 (66)	23 611 (66)	28 694 (65)

Abbreviations: AIAN, American Indian or Alaska Native; OBGYN, obstetrics and gynecology.

^a^ Sources included Transformed Medicaid Statistical Information System data from 2016,^16^ the American Medical Association Masterfile from 2016,^19^ the US Census Bureau’s American Community Survey,^24^ and the Kaiser Family Foundation (for the Medicaid expansion status of individual states in 2016).^22^

^b^ Reproductive age was 15 to 44 years.

^c^ The county in which a physician was located.

### Results From Multivariate Regression Analysis

Adjusted ORs from multivariate logistic regressions (Table 2) showed that while female family medicine physicians (OR, 1.95; 95% CI, 1.68-2.26) had higher odds of providing IUDs or implants to at least 1 Medicaid beneficiary, the same was not true for other specialties. Family medicine graduates from osteopathic medical schools (OR, 0.53; 95% CI, 0.45-0.62) had lower odds of providing IUDs or implants compared with allopathy school graduates. While family medicine IMGs had lower odds of providing IUDs or implants (OR, 0.44; 95% CI, 0.36-0.55), this pattern was not observed for other specialties. Compared with physicians younger than 35 years, family physicians in most other age groups had lower odds of providing IUDs or implants (45-54 years: OR, 0.66 [95% CI, 0.55-0.80]; 55-64 years: OR, 0.51 [95% CI, 0.39-0.65]; and 65 years or older: OR, 0.29 [95% CI, 0.19-0.44]). However, the same age groups from all other specialties had higher odds of prescribing IUDs or implants. For OBGYN physicians, compared with being younger than 35 years, being aged 35 to 44 years (OR, 3.51; 95% CI, 2.93-4.21), 45 to 54 years (OR, 3.01; 95% CI, 2.43-3.72), or 55 to 64 years (OR, 2.27; 95% CI, 1.82-2.83) was associated with higher odds of providing IUDs and implants. Practicing in a rural area was significantly negatively associated with providing IUDs or implants for both OBGYN physicians (OR, 0.49; 95% CI, 0.38-0.62) and pediatricians (OR, 0.56; 95% CI, 0.36-0.88). In terms of providing IUDs or implants, the proportion of a physician’s county population that was Black was associated with lower odds for family medicine physicians (OR, 0.98; 95% CI, 0.96-1.00), and the proportion of a physician’s county that was Asian was associated with lower odds for OBGYN physicians (OR, 0.96; 95% CI, 0.94-0.99). Conversely, the proportion of the county population that had income below the poverty line was associated with higher odds of internal medicine physicians (OR, 1.08; 95% CI, 1.02-1.14) and pediatricians (OR, 1.08; 95% CI, 1.02-1.13) providing IUDs or implants. Medicaid expansion and family planning waiver status of a physician’s state were associated with lower odds of IUD or implant provision for internal medicine physicians (Medicaid expansion: OR, 0.19 [95% CI, 0.05-0.63]; waiver status: OR, 0.12 [95% CI, 0.02-0.80]), while Medicaid expansion was associated with lower odds for pediatricians (OR, 0.28; 95% CI, 0.08-0.95).

**Table 2.** Odds of Providing at Least 1 Intrauterine Device or Implant to Medicaid Beneficiaries by Physicians in 2016

Characteristic	Odds ratio (95% CI)			
	Family medicine (n = 86 106)	Internal medicine (n = 85 095)	OBGYN (n = 35 994)	Pediatrics (n = 43 822)
**Physician characteristics**				
Female	1.95 (1.68-2.26)^a^	1.06 (0.73-1.54)	0.96 (0.91-1.02)	1.29 (0.94-1.79)
Doctor of osteopathy	0.53 (0.45-0.62)^a^	0.83 (0.51-1.35)	1.08 (0.82-1.42)	0.61 (0.41-0.91)^b^
International medical graduate	0.44 (0.36-0.55)^a^	0.73 (0.38-1.40)	1.08 (0.91-1.29)	0.67 (0.39-1.15)
Age, y				
35-44	1.10 (1.00-1.21)	1.85 (1.30-2.64)^a^	3.51 (2.93-4.21)^a^	1.90 (1.49-2.42)^a^
45-54	0.66 (0.55-0.80)^a^	2.44 (1.71-3.49)^a^	3.01 (2.43-3.72)^a^	1.80 (1.28-2.54)^a^
55-64	0.51 (0.39-0.65)^a^	2.60 (1.73-3.92)^a^	2.27 (1.82-2.83)^a^	1.83 (1.31-2.56)^a^
≥65	0.29 (0.19-0.44)^a^	1.75 (1.21-2.54)^a^	1.15 (0.94-1.41)	1.37 (0.89-2.09)
**Count**y **characteristics**^c^				
Rural	1.22 (0.95-1.57)	1.00 (0.67-1.48)	0.49 (0.38-0.62)^a^	0.56 (0.36-0.88)^b^
Log of county female population aged 15-44 y	0.99 (0.87-1.13)	1.10 (0.92-1.31)	0.78 (0.69-0.89)^a^	1.02 (0.82-1.27)
County % population below poverty line	1.01 (0.97-1.04)	1.08 (1.02-1.14)^a^	1.00 (0.97-1.03)	1.08 (1.02-1.13)^a^
Race and ethnicity				
County % population AIAN	1.00 (0.98-1.02)	0.97 (0.90-1.04)	1.00 (0.97-1.03)	0.98 (0.95-1.02)
County % population Asian	1.00 (0.97-1.02)	1.00 (0.94-1.06)	0.96 (0.94-0.99)^a^	0.99 (0.92-1.07)
County % population Black	0.98 (0.96-1.00)^b^	1.00 (0.98-1.03)	1.00 (0.98-1.01)	0.99 (0.97-1.02)
County % population Hispanic	0.99 (0.97-1.00)	0.94 (0.87-1.01)	0.99 (0.97-1.01)	0.94 (0.88-1.01)
**State policy characteristics**				
State expanded Medicaid by 2016	1.06 (0.62-1.83)	0.19 (0.05-0.63)^a^	0.84 (0.62-1.15)	0.28 (0.08-0.95)^b^
State had a Medicaid family planning waiver in 2016	0.92 (0.50-1.68)	0.12 (0.02-0.80)^b^	0.87 (0.54-1.41)	0.23 (0.04-1.33)

Abbreviations: AIAN, American Indian or Alaska Native; OBGYN, obstetrics and gynecology.

^a^ *P* < .01.

^b^ *P* < .05.

^c^ The county in which a physician was located.

Similar trends were observed for prescribing hormonal contraception to at least 1 Medicaid beneficiary (Table 3). For physicians from all specialties, being female was generally associated with higher odds of prescribing hormonal contraception to Medicaid beneficiaries. For all specialties except OBGYN, graduating from osteopathic schools and being an IMG were associated with lower odds of prescribing hormonal contraception to at least 1 Medicaid beneficiary. Except for those specializing in OBGYN, being an IMG was associated with lower odds of providing hormonal contraception (family medicine IMGs: OR, 0.80 [95% CI, 0.73-0.88]; internal medicine IMGs: OR, 0.85 [95% CI, 0.77-0.93]; and pediatric IMGs: OR, 0.85 [95% CI, 0.78-0.93]). Family physicians in all age groups older than 35 years had lower odds of prescribing hormonal contraception, but the same age groups among pediatricians and internal medicine physicians had higher odds of prescribing hormonal contraception. Rural OBGYN physicians (OR, 0.60; 95% CI, 0.48-0.76) had lower odds of prescribing hormonal contraception to Medicaid beneficiaries, but the reverse was true of internal medicine physicians (OR, 1.54; 95% CI, 1.27-1.88). The percentage of the population that was below the poverty line was associated with somewhat higher odds of physicians from all specialties prescribing hormonal contraception. The Medicaid expansion status of a state was not associated with this outcome for OBGYN physicians and pediatricians but was significantly associated for family medicine (OR, 1.50; 95% CI, 1.06-2.12) and internal medicine (OR, 1.71; 95% CI, 1.18-2.48) physicians.

**Table 3.** Odds of Prescribing at Least 1 Pill, Patch, or Ring to Medicaid Beneficiaries by Physicians in 2016

Characteristic	Odds ratio (95% CI)			
	Family medicine (n = 86 106)	Internal medicine (n = 85 095)	OBGYN (n = 35 994)	Pediatrics (n = 43 822)
**Physician characteristics**				
Female	1.80 (1.68-1.93)^a^	1.94 (1.75-2.15)^a^	1.22 (1.12-1.34)^a^	1.71 (1.55-1.89)^a^
Doctor of osteopathy	0.88 (0.78-0.98)^b^	0.88 (0.81-0.96)^a^	1.21 (0.91-1.59)	0.87 (0.79-0.95)^a^
International medical graduate	0.80 (0.73-0.88)^a^	0.85 (0.77-0.93)^a^	1.14 (1.01-1.28)^b^	0.85 (0.78-0.93)^a^
Age, y				
35-44	0.82 (0.70-0.96)^b^	1.39 (1.22-1.59)^a^	1.14 (0.88-1.47)	1.43 (1.29-1.57)^a^
45-54	0.68 (0.59-0.79)^a^	2.01 (1.70-2.36)^a^	1.28 (0.97-1.70)	1.36 (1.19-1.55)^a^
55-64	0.58 (0.51-0.67)^a^	2.08 (1.82-2.36)^a^	1.13 (0.89-1.45)	1.24 (1.07-1.44)^a^
≥65	0.41 (0.34-0.50)^a^	1.5 (1.31-1.77)2^a^	0.72 (0.55-0.94)^b^	0.90 (0.77-1.05)
**County characteristics** ^c^				
Rural	1.17 (0.99-1.39)	1.54 (1.27-1.88)^a^	0.60 (0.48-0.76)^a^	1.08 (0.87-1.34)
Log of county female population aged 15-44 y	1.03 (0.97-1.10)	1.10 (1.01-1.19)^b^	0.88 (0.81-0.96)^a^	0.94 (0.87-1.02)
County % population below poverty line	1.02 (1.00-1.04)^b^	1.03 (1.01-1.05)^a^	1.03 (1.01-1.04)^a^	1.04 (1.02-1.07)^a^
Race and ethnicity				
County % population AIAN	0.99 (0.98-1.00)^b^	0.99 (0.97-1.01)	0.99 (0.97-1.01)	0.99 (0.97-1.00)^b^
County % population Asian	1.00 (0.99-1.01)	1.01 (0.99-1.02)	1.00 (0.98-1.01)	0.99 (0.98-1.00)
County % population Black	0.99 (0.98-0.99)^a^	0.99 (0.99-1.00)^b^	0.99 (0.99-1.00)^b^	0.98 (0.97-0.99)^a^
County % population Hispanic	1.00 (0.99-1.00)	1.00 (0.99-1.01)	1.00 (0.99-1.01)	0.99 (0.99-1.00)
**State policy characteristics**				
State expanded Medicaid by 2016	1.50 (1.06-2.12)^b^	1.71 (1.18-2.48)^a^	1.21 (0.92-1.60)	1.15 (0.79-1.66)
State had a Medicaid family planning waiver in 2016	1.06 (0.78-1.45)	0.89 (0.63-1.26)	1.03 (0.79-1.34)	1.12 (0.76-1.65)

Abbreviations: AIAN, American Indian or Alaska Native; OBGYN, obstetrics and gynecology.

^a^ *P* < .01.

^b^ *P* < .05.

^c^ The county in which a physician was located.

Average marginal effects from regression models for outcomes associated with the total number of beneficiaries who were provided IUDs or implants (Table 4) and prescribed hormonal contraception (Table 5) showed that among OBGYN physicians, being a female was associated with having approximately 2 fewer beneficiaries (−2.10 beneficiaries; 95% CI, −3.28 to −0.91 beneficiaries) provided IUDs or implants (Table 4) and approximately 5 fewer beneficiaries (−5.32 beneficiaries; 95% CI, −7.48 to −3.15 beneficiaries) prescribed hormonal contraception (Table 5). In contrast, being a female physician had differing directions of associations with family medicine physicians providing IUDs or implants (AME, 0.66 beneficiaries; 95% CI, 0.42-0.91 beneficiaries) (Table 4) and prescribing hormonal contraception (AME, 2.90 beneficiaries; 95% CI, 2.26-3.55 beneficiaries) (Table 5). For OBGYN physicians, practicing in a rural county was associated with having approximately 7 fewer beneficiaries (−7.27 beneficiaries; 95% CI, −10.15 to −4.38 beneficiaries) prescribed hormonal contraception (Table 5) and 4 fewer beneficiaries (−3.91 beneficiaries; 95% CI, –5.35 to –2.48 beneficiaries) provided with IUDs or implants (Table 4). However, family medicine physicians in rural areas prescribed hormonal contraception to 1.44 additional beneficiaries (95% CI, 0.36-2.51 beneficiaries) (Table 5). State Medicaid expansion by 2016 or prior was significantly positively associated with having 12.17 additional beneficiaries (95% CI, 6.95-17.38 beneficiaries) provided hormonal contraception by OBGYN physicians and 1.92 additional beneficiaries (95% CI, 0.69-3.16 beneficiaries) provided hormonal contraception by family medicine physicians (Table 5).

**Table 4.** Average Marginal Effects of Physician Characteristics on Total Number of Medicaid Beneficiaries Provided Intrauterine Devices or Implants in 2016

Characteristic	Average marginal effect (95% CI)			
	Family medicine (n = 86 106)	Internal medicine (n = 85 095)	OBGYN (n = 35 994)	Pediatrics (n = 43 822)
**Physician characteristics**				
Female	0.66(0.42 to 0.91)^a^	0.05 (−0.06 to 0.16)	−2.10 (−3.28 to −0.91)^a^	0.54 (−8.33 to 9.40)
Doctor of osteopathy	−0.34 (−0.51 to −0.17)^a^	−0.07 (−0.20 to 0.06)	0.02 (−1.61 to 1.66)	−0.32 (−5.60 to 4.97)
International medical graduate	−0.42 (−0.55 to −0.30)^a^	−0.10 (−0.32 to 0.12)	−0.95 (−2.89 to 0.99)	−0.57 (−10.07 to 8.94)
Age, y				
35-44	0.16 (0.07 to 0.25)^a^	0.32 (−0.38 to 1.02)	9.68 (6.80 to 12.56)^a^	0.79 (−11.96 to 13.54)
45-54	−0.05 (−0.16 to 0.06)	0.39 (−0.40 to 1.17)	7.25 (4.68 to 9.83)^a^	1.67 (−27.04 to 30.38)
55-64	−0.22 (−0.35 to −0.09)^a^	0.42 (−0.49 to 1.34)	4.26 (1.23 to 7.29)^a^	1.83 (−28.92 to 32.57)
≥65	−0.45 (−0.61 to −0.29)^a^	0.04 (−0.06 to 0.14)	1.90 (−5.67 to 9.47)	0.82 (−12.46 to 14.09)
**County characteristics** ^b^				
Rural	−0.08 (−0.33 to 0.17)	−0.02 (−0.11 to 0.07)	−3.91 (−5.35 to −2.48)^a^	−1.87 (−38.53 to 34.80)
Log of county female population aged 15-44 y	−0.09 (−0.22 to 0.04)	−0.00 (−0.04 to 0.03)	−2.07 (−3.13 to −1.01)^a^	−0.13 (−2.29 to 2.03)
County % population below poverty line	0.03 (−0.01 to 0.06)	0.01 (−0.01 to 0.03)	0.22 (0.00 to 0.44)^c^	0.10 (−1.58 to 1.78)
Race and ethnicity				
County % population AIAN	0.00 (−0.02 to 0.02)	0.00 (−0.01 to 0.00)	−0.01 (−0.24 to 0.21)	0.05 (−0.90 to 0.99)
County % population Asian	0.05 (0.02 to 0.07)^a^	0.00 (0.00 to 0.01)	−0.02 (−0.15 to 0.11)	0.02 (−0.30 to 0.33)
County % population Black	−0.01 (−0.02 to 0.00)	0.00 (−0.01 to 0.00)	−0.06 (−0.14 to 0.02)	−0.02 (−0.41 to 0.36)
County % population Hispanic	0.01 (−0.01 to 0.03)	0.00 (−0.01 to 0.01)	0.07 (−0.01 to 0.14)	−0.03 (−0.53 to 0.47)
**State policy characteristics**				
State expanded Medicaid by 2016	0.08 (−0.50 to 0.66)	−0.18 (−0.65 to 0.30)	−0.08 (−2.28 to 2.13)	−0.55 (−9.30 to 8.20)
State had a Medicaid family planning waiver in 2016	0.17 (−0.33 to 0.66)	−0.01 (−0.17 to 0.16)	1.12 (−1.51 to 3.74)	−0.30 (−5.48 to 4.88)

Abbreviations: AIAN, American Indian or Alaska Native; OBGYN, obstetrics and gynecology.

^a^ *P* < .01.

^b^ The county in which a physician was located.

^c^ *P* < .05.

**Table 5.** Average Marginal Effects of Physician Characteristics on Total Number of Medicaid Beneficiaries Prescribed a Pill, Patch, or Ring in 2016

Characteristic	Average marginal effect (95% CI)			
	Family medicine (n = 86 106)	Internal medicine (n = 85 095)	OBGYN (n = 35 994)	Pediatrics (n = 43 822)
**Physician characteristics**				
Female	2.90 (2.26 to 3.55)^a^	0.87 (0.65 to 1.09)^a^	−5.32 (−7.48 to −3.15)^a^	1.35 (0.98 to 1.72)^a^
Doctor of osteopathy	−0.30 (−0.72 to 0.11)	−0.03 (−0.20 to 0.14)	7.22 (4.32 to 10.13)^a^	−0.21 (−0.48 to 0.05)
International medical graduate	0.18 (−0.61 to 0.98)	−0.01 (−0.16 to 0.13)	12.13 (7.91 to 16.35)^a^	0.26 (−0.34 to 0.87)
Age, y				
35-44	0.72 (0.31 to 1.14)^a^	0.81 (0.57 to 1.06)^a^	17.38 (12.56 to 22.20)^a^	1.69 (1.05 to 2.33)^a^
45-54	0.32 (−0.11 to 0.75)	1.34 (1.00 to 1.69)^a^	19.47 (13.00 to 25.94)^a^	2.05 (1.38 to 2.72)^a^
55-64	−0.22 (−0.65 to 0.22)	1.42 (0.93 to 1.90)^a^	16.09 (10.01 to 22.18)^a^	2.28 (1.33 to 3.23)^a^
≥65 y	−0.69 (−1.54 to 0.17)	1.25 (0.54 to 1.96)^a^	10.36 (4.67 to 16.06)^a^	1.69 (0.75 to 2.63)^a^
**County characteristics** ^b^				
Rural	1.44 (0.36 to 2.51)^a^	0.36 (−0.09 to 0.81)	−7.27 (−10.15 to −4.38)^a^	0.58 (−0.27 to 1.44)
Log of county female population aged 15-44 y	0.23 (−0.10 to 0.57)	0.07 (−0.06 to 0.20)	−3.25 (−4.65 to −1.85)^a^	0.05 (−0.24 to 0.34)
County % population below poverty line	0.17 (0.08 to 0.25)^a^	0.06 (0.03 to 0.09)^a^	0.96 (0.55 to 1.36)^a^	0.16 (0.10 to 0.21)^a^
Race and ethnicity				
County % population AIAN	−0.07 (−0.12 to −0.03)^a^	−0.03 (−0.04 to −0.01)^a^	−0.44 (−0.84 to −0.03)^c^	−0.05 (−0.10 to −0.01)^c^
County % population Asian	0.03 (−0.02 to 0.08)	0.01 (0.00 to 0.03)	−0.09 (−0.39 to 0.21)	−0.04 (−0.07 to −0.01)^a^
County % population Black	−0.03 (−0.07 to 0.01)	−0.01 (−0.01 to 0.00)	−0.08 (−0.22 to 0.05)	−0.04 (−0.07 to −0.02)^a^
County % population Hispanic	0.02 (−0.03 to 0.07)	0.00 (−0.02 to 0.02)	0.05 (−0.08 to 0.19)	−0.03 (−0.05 to 0.00)^c^
**State policy characteristics**				
State Expanded Medicaid by 2016	1.92 (0.69 to 3.16)^a^	0.55 (0.19 to 0.90)^a^	12.17 (6.95 to 17.38)^a^	0.36 (−0.55 to 1.28)
State had a Medicaid family planning waiver in 2016	0.49 (−0.82 to 1.80)	−0.12 (−0.56 to 0.33)	0.83 (−4.53 to 6.20)	0.28 (−0.72 to 1.28)

Abbreviations: AIAN, American Indian or Alaska Native; OBGYN, obstetrics and gynecology.

^a^ *P* < .01.

^b^ The county in which a physician was located.

^c^ *P* < .05.

## Discussion

In this cross-sectional study of the Medicaid contraceptive care workforce in 2016, we found that physician characteristics, including age, sex, specialty, medical training, and rural location, and the socioeconomic conditions of a physician’s county were associated with both providing any contraceptive care and the total number of beneficiaries provided contraceptive care. Results from descriptive analysis provide first glimpses, to our knowledge, of how physicians engage in reproductive health–related services in state Medicaid programs. Of the physicians in the sample, 48% prescribed hormonal birth control methods, while 10% provided IUDs or implants. A previous report^26^ showed that about 5% of women aged 21 to 44 years who were covered by Medicaid and at risk of pregnancy received IUDs or implants, and about 25% of this group received hormonal contraception. While patient preferences may have influenced some of these differences, there was a large proportion of Medicaid-participating primary care clinicians who provided hormonal contraception but not IUDs or implants. For Medicaid beneficiaries seeking these services, a primary care clinician who needs to refer them to someone else can be an additional barrier to access.

We found a wide variation in contraception provision by individual specialties. These differences reflect the variations in specialties’ target populations and their revenue reliance on Medicaid. However, these findings also suggest that there is opportunity for increased engagement in contraceptive services by certain specialties. It is important to note that the demand for OBGYN physicians is projected to outpace supply as early as 2031.^27^ Thus, increasing the provision of contraceptive services across all primary care specialties will be important to meet the demand for these services in the future.

Results from regression analyses showed that female physicians from most specialties had higher odds of providing contraceptive services and provided these services to a higher number of Medicaid beneficiaries. This is consistent with prior literature on general Medicaid participation of primary care physicians.^28^ This may also reflect patient preference for female physicians due to the perception that these physicians have personal knowledge of contraception and indicate patients having higher comfort levels discussing contraception with female physicians.^29,30,31^

International medical graduates from nearly all specialties except OBGYN had lower odds of providing contraceptive services, but the proportion of IMGs among OBGYN physicians is a fraction of that in other specialties.^32^ In addition, compared with their colleagues trained in US medical schools, IMGs are more likely to practice in areas that have physician shortages,^33,34^ and they generally see a higher proportion of Medicaid beneficiaries.^35^ Policy makers should take note of these findings since the growth in the number of IMGs has outpaced that of US medical graduates in recent years.^36^

Younger physicians had higher odds of providing both hormonal contraception and IUDs or implants except in the case of family medicine physicians. It is possible that younger physicians have more knowledge and training and are less likely to hold negative beliefs about modern contraceptive methods such as IUDs and implants.^37^ Studies have shown that younger physicians are more likely to initiate conversations about contraceptive care with their patients and are more likely to offer IUDs and implants.^38,39^ Finally, as younger physicians are more likely to see Medicaid patients, our results may reflect the additive impact of these issues.

Several community-level factors were associated with contraceptive service provision. The percentage of the population that was below the poverty line was associated with somewhat higher odds of physicians from all specialties prescribing hormonal contraception. Family medicine and OBGYN physicians in counties with a higher percentage of Black individuals had lower odds of prescribing hormonal contraception. These findings could be related to patient preferences. However, given the increasing evidence of clinician biases in health care,^40^ these findings need further research to understand the underlying mechanisms.

Finally, we found that state policy characteristics were associated with contraceptive service provision. Belonging to a state that expanded Medicaid before 2016 was associated with significantly higher odds of prescribing hormonal contraception for physicians from certain specialties. Having a Medicaid family planning waiver in a state was generally not associated with physicians’ contraceptive service provision. A previous study^11^ showed that nearly one-third of women in newly Medicaid-eligible populations received contraceptive services. Since Medicaid expansion is associated with an increase in the odds of physicians’ providing IUDs or implants, this may suggest an additional barrier related to these contraception services.

Understanding contraception provision by physicians has become more important in the context of recent developments in state policies on access to abortion. Over the next few years, medical students’ training on this subject is expected to be constrained by state laws on abortions.^41^ In the future, physicians may have to optimize how they prescribe contraception (by prescribing it for longer durations), assess the possibility of contraception failure among its users, and recommend the use of tools such as app-based reminders or alarms.^42^ Additionally, since a relatively smaller proportion of physicians provide contraceptives, more physicians and medical students may have to undergo training for effective, long-acting, reversible contraception methods such as IUDs.^42^

Many states’ recent expansion of Medicaid coverage for up to 1 year post partum will make contraception more accessible for a large group of beneficiaries.^43^ Early evidence showed that such coverage expansion in Texas was associated with substantially higher utilization of contraceptive services.^44^ This may also positively impact physicians’ contraception provision in such states.

### Limitations

There are several limitations to our analysis. First, there are limitations of the data used. Substantial variation existed in the quality of T-MSIS data submitted by states.^45^ We used data from 44 states and Puerto Rico (eTables 4 and 5 and eFigures 2 and 3 in Supplement 1), for which programs covered approximately 86% of all Medicaid beneficiaries in 2016.^46^ We used data from 2016, which are somewhat dated. Due to data quality issues, we did not use information about physicians’ patient panels. We did not analyze any claims that appeared exclusively in the T-MSIS inpatient file since such claims do not identify the individual practitioner who provided the service. We may have therefore missed the provision of some contraceptive services. We did not control for the percentage of a county’s population that belonged to the “other” race category. This category may have included those who self-reported as belonging to more than 1 race (≥2).^24^ Second, our analysis did not include any information about the practices in which physicians operated. Several practice-level factors, such as ownership structure, size, and location, may impact physicians’ willingness to provide contraceptive care to Medicaid beneficiaries.^28^ Third, our method of assigning states to physicians could have influenced the findings on state policy characteristics. However, we reassigned states of only 8% (18 978 of 251 017) of physicians in the sample (eFigures 4 and 5 in Supplement 1). Finally, we did not include nonphysicians in our analysis (nurse practitioners and physician assistants) since we did not have complete information about their individual-level characteristics.

## Conclusions

This cross-sectional study offers, to our knowledge, the first national-level assessment of how individual physician- and community-level characteristics are associated with contraceptive service provision to Medicaid beneficiaries. We found that physician characteristics, including age, sex, specialty, medical training, and rural location, and the socioeconomic conditions of a physician’s county were associated with both providing any contraceptive care and the total number of beneficiaries provided contraceptive care. These findings varied across clinical specialties; thus, policies tailored for different physician types are essential to ensure that Medicaid beneficiaries have access to contraception.

## Notes

Supplement 1. **eFigure 1.** Physician Sample Selection Flow Diagram **eTable 1.** List of Codes Used in the Study **eTable 2.** Demographic Characteristics of Physicians in the Sample That Provided IUDs or Implants **eTable 3.** Demographic Characteristics of Physicians in the Sample Prescribing at Least 1 Pill, Patch, or Ring **eTable 4.** Multivariate Regression Results for the Full Analytical Sample (N = 251 017) **eFigure 2.** Overview of State Selection Process **eTable 5.** T-MSIS DQ Atlas Single Topic Summary **eFigure 3.** An Example of Filling in NPIs in T-MSIS Encounter Claims **eTable 6.** State Data Quality Assessment and Sample Selection **eFigure 4.** Physician State Reassignment Process Example **eFigure 5.** Physician State Reassignment Details

Supplement 2.
Data Sharing Statement

## References

[1] Blumenthal PD, Voedisch A, Gemzell-Danielsson K. Strategies to prevent unintended pregnancy: increasing use of long-acting reversible contraception. Hum Reprod Update. 2011;17(1):121-137. doi:10.1093/humupd/dmq026 20634208

[2] Sutton MY, Anachebe NF, Lee R, Skanes H. Racial and ethnic disparities in reproductive health services and outcomes, 2020. Obstet Gynecol. 2021;137(2):225-233. doi:10.1097/AOG.0000000000004224 33416284 PMC7813444

[3] Beroukhim G, Mahabamunuge J, Pal L. Racial disparities in access to reproductive health and fertility care in the United States. Curr Opin Obstet Gynecol. 2022;34(3):138-146. doi:10.1097/GCO.0000000000000780 35645012

[4] Rodriguez MI, Meath T, Watson K, Daly A, Tracy K, McConnell KJ. Postpartum contraceptive use among US Medicaid recipients. JAMA Netw Open. 2022;5(1):e2145175-e2145175. doi:10.1001/jamanetworkopen.2021.45175 35080605 PMC8792884

[5] Swan LET, Vu H, Higgins JA, Bui LM, Malecki K, Green TL. Exploring financial stress and resource deprivation as barriers to preferred contraceptive use in Wisconsin in 2021. Contraception. 2022;115:22-26. doi:10.1016/j.contraception.2022.07.014 35940300

[6] Sommers BD, Kronick R. Measuring Medicaid physician participation rates and implications for policy. J Health Polit Policy Law. 2016;41(2):211-224. doi:10.1215/03616878-3476117 26732320

[7] Spivack SB, Murray GF, Rodriguez HP, Lewis VA. Avoiding Medicaid: characteristics of primary care practices with no Medicaid revenue. Health Aff (Millwood). 2021;40(1):98-104. doi:10.1377/hlthaff.2020.00100 33400572 PMC9924217

[8] Holgash K, Heberlein M. Physician acceptance of new Medicaid patients: what matters and what doesn’t. *Health Affairs*. April 10, 2019. Accessed October 4, 2022. https://www.healthaffairs.org/do/10.1377/forefront.20190401.678690

[9] Oostrom T, Einav L, Finkelstein A. Outpatient office wait times and quality of care for Medicaid patients. Health Aff (Millwood). 2017;36(5):826-832. doi:10.1377/hlthaff.2016.1478 28461348 PMC5812017

[10] Ranji U, Gomez I, Salganicoff A, Rosenzweig C, Kellenberg R, Gifford K. Medicaid coverage of family planning benefits: findings from a 2021 state survey. Kaiser Family Foundation. February 17, 2022. Accessed October 4, 2022. https://www.kff.org/womens-health-policy/report/medicaid-coverage-of-family-planning-benefits-findings-from-a-2021-state-survey/

[11] Gibbs SE, Harvey SM, Larson A, Yoon J, Luck J. Contraceptive services after Medicaid expansion in a state with a Medicaid family planning waiver program. J Womens Health (Larchmt). 2021;30(5):750-757. doi:10.1089/jwh.2020.8351 33085917

[12] Chen C, Strasser J, Banawa R, . Who is providing contraception care in the United States? an observational study of the contraception workforce. Am J Obstet Gynecol. 2022;226(2):232.e1-232.e11. doi:10.1016/j.ajog.2021.08.015 34418348

[13] Nisen MB, Peterson LE, Cochrane A, Rubin SE. US family physicians’ intrauterine and implantable contraception provision: results from a national survey. Contraception. 2016;93(5):432-437. doi:10.1016/j.contraception.2016.01.004 26776938 PMC4842114

[14] Davis SA, Braykov NP, Lathrop E, Haddad LB. Familiarity with long-acting reversible contraceptives among obstetrics and gynecology, family medicine, and pediatrics residents: results of a 2015 national survey and implications for contraceptive provision for adolescents. J Pediatr Adolesc Gynecol. 2018;31(1):40-44. doi:10.1016/j.jpag.2017.09.007 28942106

[15] Kaskowitz A, Quint E, Zochowski M, Caldwell A, Vinekar K, Dalton VK. Contraception delivery in pediatric and specialist pediatric practices. J Pediatr Adolesc Gynecol. 2017;30(2):184-187. doi:10.1016/j.jpag.2015.10.022 26626787

[16] Centers for Medicare & Medicaid Services. Transformed Medicaid Statistical Information System (T-MSIS), Release 2. Accessed October 4, 2022. https://www.medicaid.gov/medicaid/data-systems/macbis/transformed-medicaid-statistical-information-system-t-msis/index.html

[17] US Dept of Health and Human Services; Office of the Assistant Secretary for Health; Office of Population Affairs. Contraceptive provision measures. Accessed October 4, 2022. https://opa.hhs.gov/claims-data-sas-program-instructions

[18] National Plan and Provider Enumeration System; US Dept of Health and Human Services; Centers for Medicare & Medicaid Services. January 2017. Accessed October 4, 2022. https://nppes.cms.hhs.gov/#/

[19] American Medical Association. AMA Physician Masterfile. Accessed October 4, 2022. https://www.ama-assn.org/about/masterfile/ama-physician-masterfile

[20] Health Resources and Services Administration; US Dept of Health and Human Services. Area health resources files. Accessed October 4, 2022. https://data.hrsa.gov/topics/health-workforce/ahrf

[21] US Census Bureau. Data profiles. Accessed October 4, 2022. https://www.census.gov/acs/www/data/data-tables-and-tools/data-profiles/2016/

[22] Kaiser Family Foundation. Status of state Medicaid expansion decisions: interactive map. November 9, 2022. Accessed October 4, 2022. https://www.kff.org/medicaid/issue-brief/status-of-state-medicaid-expansion-decisions-interactive-map/

[23] US Dept of Agriculture. Economic Research Service. Rural-Urban Continuum Codes. Accessed October 4, 2022. https://www.ers.usda.gov/data-products/rural-urban-continuum-codes.aspx

[24] US Census Bureau. Methodology for the United States population estimates: vintage 2019, Version 2. March 2020. Accessed January 7, 2023. https://www2.census.gov/programs-surveys/popest/technical-documentation/methodology/2010-2019/natstcopr-methv2.pdf

[25] *Stata Statistical Software*. Computer program. Release 17. StataCorp LLC. Accessed October 4, 2022. https://www.stata.com/

[26] Centers for Medicare & Medicaid Services; Medicaid & CHIP. Health Care Quality Measures—quality of maternal and perinatal health care in Medicaid and CHIP: findings from the 2020 Maternity Core Set. November 2021. Accessed October 4, 2022. https://www.medicaid.gov/medicaid/quality-of-care/downloads/2021-maternity-chart-pack.pdf

[27] US Dept of Health and Human Services; Health Resources and Services Administration; Bureau of Health Workforce; National Center for Health Workforce Analysis. Projections of supply and demand for women’s health service providers: 2018-2030. March 2021. Accessed October 4, 2022. https://bhw.hrsa.gov/sites/default/files/bureau-health-workforce/data-research/projections-supply-demand-2018-2030.pdf

[28] Tipirneni R, Kieffer EC, Ayanian JZ, . Factors influencing primary care providers’ decisions to accept new Medicaid patients under Michigan’s Medicaid expansion. Am J Manag Care. 2019;25(3):120-127. 30875180 PMC7169442

[29] Fox E, Reyna A, Malcolm NM, . Client preferences for contraceptive counseling: a systematic review. Am J Prev Med. 2018;55(5):691-702. doi:10.1016/j.amepre.2018.06.006 30342632 PMC6655529

[30] Becker D, Klassen AC, Koenig MA, LaVeist TA, Sonenstein FL, Tsui AO. Women’s perspectives on family planning service quality: an exploration of differences by race, ethnicity and language. Perspect Sex Reprod Health. 2009;41(3):158-165. doi:10.1363/4115809 19740233

[31] Löwe P. Embodied expertise: women’s perceptions of the contraception consultation. Health (London). 2005;9(3):361-378. doi:10.1177/1363459305052906 15937037

[32] Association of American Medical Colleges. Physician specialty data report: ACGME residents and fellows who are international medical graduates (IMGs) by specialty, 2017. Accessed October 8, 2022. https://www.aamc.org/data-reports/workforce/interactive-data/acgme-residents-and-fellows-who-are-international-medical-graduates-imgs-specialty-2017

[33] Torrey EF, Torrey BB. The US distribution of physicians from lower income countries. PLoS One. 2012;7(3):e33076. doi:10.1371/journal.pone.0033076 22457735 PMC3310056

[34] Leng SW, Opalek A. International medical graduates’ contributions to the United States health workforce. Foundation for Advancement of International Medical Education and Research. July 2017. Accessed October 8, 2022. https://www.researchgate.net/publication/318377778_International_Medical_Graduates%27_Contributions_to_the_United_States_Health_Workforce

[35] Hing E, Lin S. Role of international medical graduates providing office-based medical care: United States, 2005-2006. NCHS Data Brief. 2009;(13):1-8. 19389325

[36] American Medical Association. How IMGs have changed the face of American medicine. October 19, 2021. Accessed October 8, 2022. https://www.ama-assn.org/education/international-medical-education/how-imgs-have-changed-face-american-medicine

[37] Akers AY, Gold MA, Borrero S, Santucci A, Schwarz EB. Providers’ perspectives on challenges to contraceptive counseling in primary care settings. J Womens Health (Larchmt). 2010;19(6):1163-1170. doi:10.1089/jwh.2009.1735 20420508 PMC2940510

[38] Dehlendorf C, Levy K, Ruskin R, Steinauer J. Health care providers’ knowledge about contraceptive evidence: a barrier to quality family planning care? Contraception. 2010;81(4):292-298. doi:10.1016/j.contraception.2009.11.006 20227544 PMC2892417

[39] Harper CC, Blum M, de Bocanegra HT, . Challenges in translating evidence to practice: the provision of intrauterine contraception. Obstet Gynecol. 2008;111(6):1359-1369. doi:10.1097/AOG.0b013e318173fd83 18515520

[40] Morden NE, Chyn D, Wood A, Meara E. Racial inequality in prescription opioid receipt—role of individual health systems. N Engl J Med. 2021;385(4):342-351. doi:10.1056/NEJMsa2034159 34289277 PMC8402927

[41] Traub AM, Mermin-Bunnell K, Pareek P, . The implications of overturning *Roe v. Wade* on medical education and future physicians. Lancet Reg Health Am. 2022;14:100334. doi:10.1016/j.lana.2022.100334 36777391 PMC9904004

[42] Bernard R. Responding to the overturning of Roe v. Wade: 6 immediate actions for primary care internists. J Gen Intern Med. 2023;38(1):219-220. doi:10.1007/s11606-022-07869-8 36323821 PMC9849600

[43] Kaiser Family Foundation. Medicaid postpartum coverage extension tracker. January 27, 2023. Accessed January 27, 2023. https://www.kff.org/medicaid/issue-brief/medicaid-postpartum-coverage-extension-tracker/

[44] Wang X, Pengetnze YM, Eckert E, Keever G, Chowdhry V. Extending postpartum Medicaid beyond 60 days improves care access and uncovers unmet needs in a Texas Medicaid health maintenance organization. Front Public Health. 2022;10:841832. doi:10.3389/fpubh.2022.841832 35592081 PMC9110670

[45] Centers for Medicare & Medicaid Services. DQAtlas. Accessed October 4, 2022. https://www.medicaid.gov/dq-atlas/welcome

[46] Kaiser Family Foundation. Total monthly Medicaid & CHIP enrollment and pre-ACA enrollment. September 2022. Accessed January 7, 2023. https://www.kff.org/health-reform/state-indicator/total-monthly-medicaid-and-chip-enrollment/

