# Supplementary material for: Association of Primary Care Physicians’ Individual- and Community-Level Characteristics With Contraceptive Service Provision to Medicaid Beneficiaries
Source: JAMA Health Forum. 2023 Mar 17;4(3):e230106. doi: 10.1001/jamahealthforum.2023.0106 (PMC10024198; doi:10.1001/jamahealthforum.2023.0106)
Supplement: Supplement 1. — eFigure 1. Physician Sample Selection Flow Diagram eTable 1. List of Codes Used in the Study eTable 2. Demographic Characteristics of Physicians in the Sample That Provided IUDs or Implants eTable 3. Demographic Characteristics of Physicians in the Sample Prescribing at Least 1 Pill, Patch, or Ring eTable 4. Multivariate Regression Results for the Full Analytical Sample (N = 251 017) eFigure 2. Overview of State Selection Process eTable 5. T-MSIS DQ Atlas Single Topic Summary eFigure 3. An Example of Filling in NPIs in T-MSIS Encounter Claims eTable 6. State Data Quality Assessment and Sample Selection eFigure 4. Physician State Reassignment Process Example eFigure 5. Physician State Reassignment Details [file jamahealthforum-e230106-s001.pdf]

Supplemental Online Content

Bodas M, Strasser J, Luo Q, Schenk E, Chen C. Association of primary care physicians' individual- and community-level characteristics with contraceptive service provision to Medicaid beneficiaries. *JAMA Health Forum*. 2023;4(3):e230106. doi:10.1001/jamahealthforum.2023.0106

eFigure 1. Physician Sample Selection Flow Diagram

eTable 1. List of Codes Used in the Study

eTable 2. Demographic Characteristics of Physicians in the Sample That Provided IUDs or Implants

eTable 3. Demographic Characteristics of Physicians in the Sample Prescribing at Least 1 Pill, Patch, or Ring

eTable 4. Multivariate Regression Results for the Full Analytical Sample (N = 251 017)

eFigure 2. Overview of State Selection Process

eTable 5. T-MSIS DQ Atlas Single Topic Summary

eFigure 3. An Example of Filling in NPIs in T-MSIS Encounter Claims

eTable 6. State Data Quality Assessment and Sample Selection

eFigure 4. Physician State Reassignment Process Example

eFigure 5. Physician State Reassignment Details

This supplementary material has been provided by the authors to give readers additional information about their work.

© 2023 Bodas M et al. *JAMA Health Forum*.

eFigure 1. Physician Sample Selection Flow Diagram

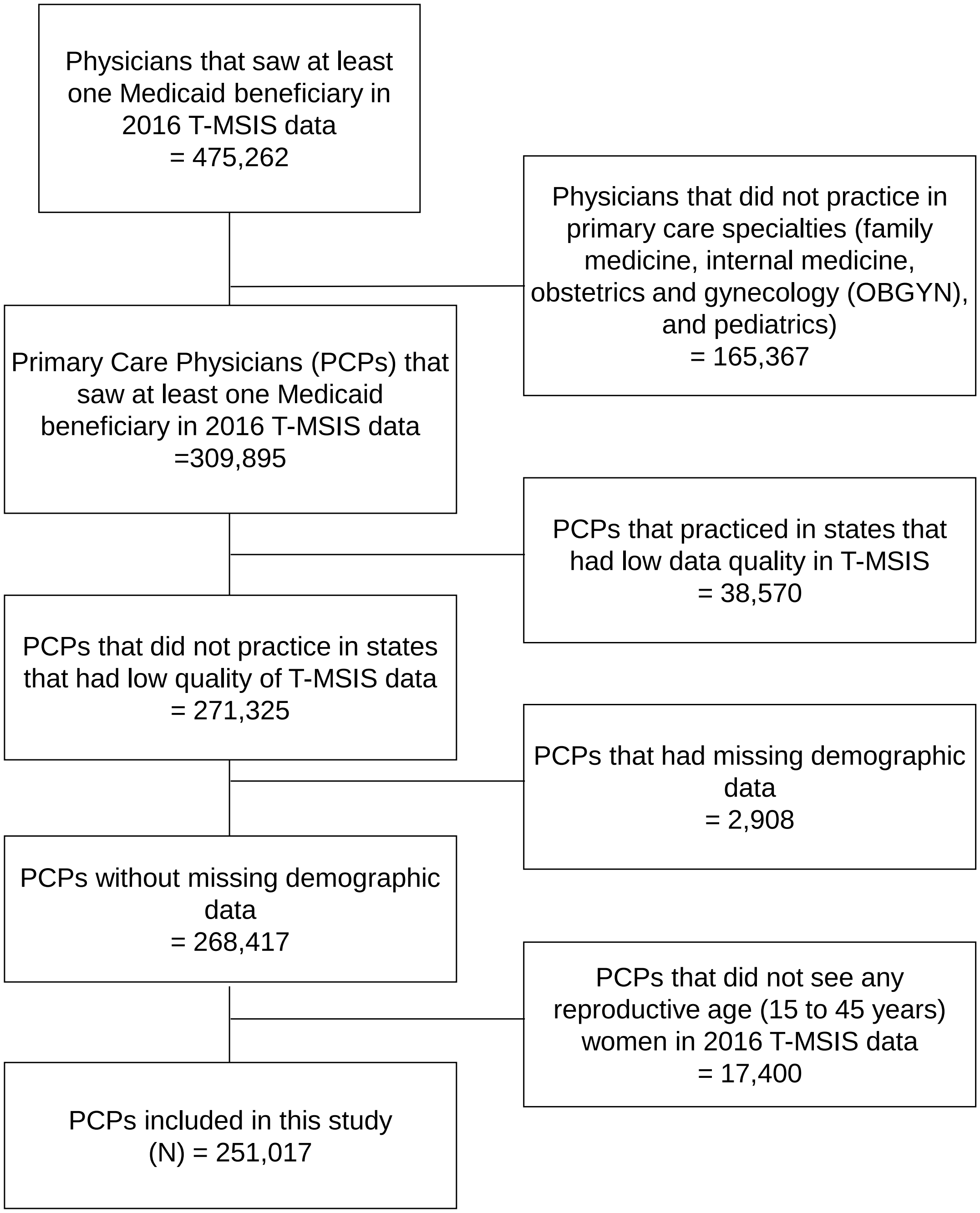

© 2023 Bodas M et al. *JAMA Health Forum*.

eTable 1. List of Codes Used in the Study

Code	Type	Description
58300	Current Procedural Terminology (CPT)	Intrauterine device (IUD) Insertion
58301	Current Procedural Terminology (CPT)	IUD Removal
J1050	Current Procedural Terminology (CPT)	Birth control shot
J1051	Current Procedural Terminology (CPT)	Birth control shot
J1055	Current Procedural Terminology (CPT)	Birth control shot
J7296	Current Procedural Terminology (CPT)	IUD Insertion/Device
J7297	Current Procedural Terminology (CPT)	IUD Insertion/Device
J7298	Current Procedural Terminology (CPT)	IUD Insertion/Device
J7300	Current Procedural Terminology (CPT)	IUD Insertion/Device
J7301	Current Procedural Terminology (CPT)	IUD Insertion/Device
J7302	Current Procedural Terminology (CPT)	IUD Insertion/Device
J7306	Current Procedural Terminology (CPT)	Contraceptive Implant
J7307	Current Procedural Terminology (CPT)	Contraceptive Implant
S4981	Current Procedural Terminology (CPT)	IUD Insertion/Device
S4989	Current Procedural Terminology (CPT)	IUD Insertion/Device
003783340	National Drug Code	Transdermal contraceptive patch
504580192	National Drug Code	Transdermal contraceptive patch
000082514	National Drug Code	Oral Contraceptive Pill
000235862	National Drug Code	Oral Contraceptive Pill
000520261	National Drug Code	Oral Contraceptive Pill
000520283	National Drug Code	Oral Contraceptive Pill
000621251	National Drug Code	Oral Contraceptive Pill
000621411	National Drug Code	Oral Contraceptive Pill
000621907	National Drug Code	Oral Contraceptive Pill
000932090	National Drug Code	Oral Contraceptive Pill
000932140	National Drug Code	Oral Contraceptive Pill
000933134	National Drug Code	Oral Contraceptive Pill
000935328	National Drug Code	Oral Contraceptive Pill
000935423	National Drug Code	Oral Contraceptive Pill
000936148	National Drug Code	Oral Contraceptive Pill
003786550	National Drug Code	Oral Contraceptive Pill
003787272	National Drug Code	Oral Contraceptive Pill
003787277	National Drug Code	Oral Contraceptive Pill
003787280	National Drug Code	Oral Contraceptive Pill
003787281	National Drug Code	Oral Contraceptive Pill
003787283	National Drug Code	Oral Contraceptive Pill
003787285	National Drug Code	Oral Contraceptive Pill
003787287	National Drug Code	Oral Contraceptive Pill
003787292	National Drug Code	Oral Contraceptive Pill
003787296	National Drug Code	Oral Contraceptive Pill

© 2023 Bodas M et al. *JAMA Health Forum*.

003787298	National Drug Code	Oral Contraceptive Pill
003787300	National Drug Code	Oral Contraceptive Pill
003787301	National Drug Code	Oral Contraceptive Pill
003787306	National Drug Code	Oral Contraceptive Pill
003787308	National Drug Code	Oral Contraceptive Pill
004300005	National Drug Code	Oral Contraceptive Pill
004300010	National Drug Code	Oral Contraceptive Pill
004300420	National Drug Code	Oral Contraceptive Pill
004300482	National Drug Code	Oral Contraceptive Pill
004300530	National Drug Code	Oral Contraceptive Pill
004300535	National Drug Code	Oral Contraceptive Pill
004300540	National Drug Code	Oral Contraceptive Pill
004300570	National Drug Code	Oral Contraceptive Pill
004300580	National Drug Code	Oral Contraceptive Pill
005550344	National Drug Code	Oral Contraceptive Pill
005550715	National Drug Code	Oral Contraceptive Pill
005559008	National Drug Code	Oral Contraceptive Pill
005559009	National Drug Code	Oral Contraceptive Pill
005559010	National Drug Code	Oral Contraceptive Pill
005559012	National Drug Code	Oral Contraceptive Pill
005559014	National Drug Code	Oral Contraceptive Pill
005559016	National Drug Code	Oral Contraceptive Pill
005559018	National Drug Code	Oral Contraceptive Pill
005559020	National Drug Code	Oral Contraceptive Pill
005559025	National Drug Code	Oral Contraceptive Pill
005559026	National Drug Code	Oral Contraceptive Pill
005559027	National Drug Code	Oral Contraceptive Pill
005559028	National Drug Code	Oral Contraceptive Pill
005559032	National Drug Code	Oral Contraceptive Pill
005559034	National Drug Code	Oral Contraceptive Pill
005559043	National Drug Code	Oral Contraceptive Pill
005559045	National Drug Code	Oral Contraceptive Pill
005559047	National Drug Code	Oral Contraceptive Pill
005559049	National Drug Code	Oral Contraceptive Pill
005559050	National Drug Code	Oral Contraceptive Pill
005559051	National Drug Code	Oral Contraceptive Pill
005559064	National Drug Code	Oral Contraceptive Pill
005559066	National Drug Code	Oral Contraceptive Pill
005559123	National Drug Code	Oral Contraceptive Pill
005559131	National Drug Code	Oral Contraceptive Pill
006033590	National Drug Code	Oral Contraceptive Pill
006037512	National Drug Code	Oral Contraceptive Pill
006037521	National Drug Code	Oral Contraceptive Pill

© 2023 Bodas M et al. *JAMA Health Forum*.

006037525	National Drug Code	Oral Contraceptive Pill
006037540	National Drug Code	Oral Contraceptive Pill
006037606	National Drug Code	Oral Contraceptive Pill
006037607	National Drug Code	Oral Contraceptive Pill
006037608	National Drug Code	Oral Contraceptive Pill
006037609	National Drug Code	Oral Contraceptive Pill
006037610	National Drug Code	Oral Contraceptive Pill
006037625	National Drug Code	Oral Contraceptive Pill
006037634	National Drug Code	Oral Contraceptive Pill
006037642	National Drug Code	Oral Contraceptive Pill
006037663	National Drug Code	Oral Contraceptive Pill
007814058	National Drug Code	Oral Contraceptive Pill
007814060	National Drug Code	Oral Contraceptive Pill
007814062	National Drug Code	Oral Contraceptive Pill
007814075	National Drug Code	Oral Contraceptive Pill
007815575	National Drug Code	Oral Contraceptive Pill
007815583	National Drug Code	Oral Contraceptive Pill
007815584	National Drug Code	Oral Contraceptive Pill
007815656	National Drug Code	Oral Contraceptive Pill
007815658	National Drug Code	Oral Contraceptive Pill
167140073	National Drug Code	Oral Contraceptive Pill
167140340	National Drug Code	Oral Contraceptive Pill
167140346	National Drug Code	Oral Contraceptive Pill
167140347	National Drug Code	Oral Contraceptive Pill
167140348	National Drug Code	Oral Contraceptive Pill
167140359	National Drug Code	Oral Contraceptive Pill
167140360	National Drug Code	Oral Contraceptive Pill
167140363	National Drug Code	Oral Contraceptive Pill
167140365	National Drug Code	Oral Contraceptive Pill
167140366	National Drug Code	Oral Contraceptive Pill
167140367	National Drug Code	Oral Contraceptive Pill
167140370	National Drug Code	Oral Contraceptive Pill
167140404	National Drug Code	Oral Contraceptive Pill
167140405	National Drug Code	Oral Contraceptive Pill
167140406	National Drug Code	Oral Contraceptive Pill
167140407	National Drug Code	Oral Contraceptive Pill
167140408	National Drug Code	Oral Contraceptive Pill
167140413	National Drug Code	Oral Contraceptive Pill
167140416	National Drug Code	Oral Contraceptive Pill
167140440	National Drug Code	Oral Contraceptive Pill
167140441	National Drug Code	Oral Contraceptive Pill
167140464	National Drug Code	Oral Contraceptive Pill
353560368	National Drug Code	Oral Contraceptive Pill

© 2023 Bodas M et al. *JAMA Health Forum*.

353560370	National Drug Code	Oral Contraceptive Pill
433860620	National Drug Code	Oral Contraceptive Pill
458020840	National Drug Code	Oral Contraceptive Pill
501020100	National Drug Code	Oral Contraceptive Pill
501020120	National Drug Code	Oral Contraceptive Pill
501020128	National Drug Code	Oral Contraceptive Pill
501020130	National Drug Code	Oral Contraceptive Pill
501020154	National Drug Code	Oral Contraceptive Pill
504190402	National Drug Code	Oral Contraceptive Pill
504190403	National Drug Code	Oral Contraceptive Pill
504190405	National Drug Code	Oral Contraceptive Pill
504190407	National Drug Code	Oral Contraceptive Pill
504190409	National Drug Code	Oral Contraceptive Pill
504190482	National Drug Code	Oral Contraceptive Pill
504190483	National Drug Code	Oral Contraceptive Pill
504580171	National Drug Code	Oral Contraceptive Pill
504580176	National Drug Code	Oral Contraceptive Pill
504580178	National Drug Code	Oral Contraceptive Pill
504580191	National Drug Code	Oral Contraceptive Pill
504580194	National Drug Code	Oral Contraceptive Pill
504580196	National Drug Code	Oral Contraceptive Pill
504580197	National Drug Code	Oral Contraceptive Pill
504580251	National Drug Code	Oral Contraceptive Pill
512850079	National Drug Code	Oral Contraceptive Pill
512850080	National Drug Code	Oral Contraceptive Pill
512850081	National Drug Code	Oral Contraceptive Pill
512850082	National Drug Code	Oral Contraceptive Pill
512850083	National Drug Code	Oral Contraceptive Pill
512850087	National Drug Code	Oral Contraceptive Pill
512850092	National Drug Code	Oral Contraceptive Pill
512850114	National Drug Code	Oral Contraceptive Pill
512850120	National Drug Code	Oral Contraceptive Pill
512850125	National Drug Code	Oral Contraceptive Pill
512850126	National Drug Code	Oral Contraceptive Pill
512850127	National Drug Code	Oral Contraceptive Pill
512850128	National Drug Code	Oral Contraceptive Pill
512850129	National Drug Code	Oral Contraceptive Pill
512850131	National Drug Code	Oral Contraceptive Pill
512850424	National Drug Code	Oral Contraceptive Pill
512850431	National Drug Code	Oral Contraceptive Pill
512850546	National Drug Code	Oral Contraceptive Pill
512850769	National Drug Code	Oral Contraceptive Pill
512850942	National Drug Code	Oral Contraceptive Pill

© 2023 Bodas M et al. *JAMA Health Forum*.

512850943	National Drug Code	Oral Contraceptive Pill
516600127	National Drug Code	Oral Contraceptive Pill
516600572	National Drug Code	Oral Contraceptive Pill
518620007	National Drug Code	Oral Contraceptive Pill
518620012	National Drug Code	Oral Contraceptive Pill
518620028	National Drug Code	Oral Contraceptive Pill
518620036	National Drug Code	Oral Contraceptive Pill
518620045	National Drug Code	Oral Contraceptive Pill
518620047	National Drug Code	Oral Contraceptive Pill
518620072	National Drug Code	Oral Contraceptive Pill
518620097	National Drug Code	Oral Contraceptive Pill
518620102	National Drug Code	Oral Contraceptive Pill
518620238	National Drug Code	Oral Contraceptive Pill
518620260	National Drug Code	Oral Contraceptive Pill
518620279	National Drug Code	Oral Contraceptive Pill
518620284	National Drug Code	Oral Contraceptive Pill
518620292	National Drug Code	Oral Contraceptive Pill
518620318	National Drug Code	Oral Contraceptive Pill
518620470	National Drug Code	Oral Contraceptive Pill
518620471	National Drug Code	Oral Contraceptive Pill
518620510	National Drug Code	Oral Contraceptive Pill
518620545	National Drug Code	Oral Contraceptive Pill
518620564	National Drug Code	Oral Contraceptive Pill
525440054	National Drug Code	Oral Contraceptive Pill
525440064	National Drug Code	Oral Contraceptive Pill
525440087	National Drug Code	Oral Contraceptive Pill
525440143	National Drug Code	Oral Contraceptive Pill
525440165	National Drug Code	Oral Contraceptive Pill
525440167	National Drug Code	Oral Contraceptive Pill
525440204	National Drug Code	Oral Contraceptive Pill
525440219	National Drug Code	Oral Contraceptive Pill
525440228	National Drug Code	Oral Contraceptive Pill
525440233	National Drug Code	Oral Contraceptive Pill
525440235	National Drug Code	Oral Contraceptive Pill
525440245	National Drug Code	Oral Contraceptive Pill
525440247	National Drug Code	Oral Contraceptive Pill
525440248	National Drug Code	Oral Contraceptive Pill
525440249	National Drug Code	Oral Contraceptive Pill
525440254	National Drug Code	Oral Contraceptive Pill
525440259	National Drug Code	Oral Contraceptive Pill
525440265	National Drug Code	Oral Contraceptive Pill
525440268	National Drug Code	Oral Contraceptive Pill
525440274	National Drug Code	Oral Contraceptive Pill

© 2023 Bodas M et al. *JAMA Health Forum*.

525440275	National Drug Code	Oral Contraceptive Pill
525440276	National Drug Code	Oral Contraceptive Pill
525440279	National Drug Code	Oral Contraceptive Pill
525440287	National Drug Code	Oral Contraceptive Pill
525440290	National Drug Code	Oral Contraceptive Pill
525440291	National Drug Code	Oral Contraceptive Pill
525440292	National Drug Code	Oral Contraceptive Pill
525440295	National Drug Code	Oral Contraceptive Pill
525440298	National Drug Code	Oral Contraceptive Pill
525440383	National Drug Code	Oral Contraceptive Pill
525440384	National Drug Code	Oral Contraceptive Pill
525440550	National Drug Code	Oral Contraceptive Pill
525440552	National Drug Code	Oral Contraceptive Pill
525440554	National Drug Code	Oral Contraceptive Pill
525440629	National Drug Code	Oral Contraceptive Pill
525440630	National Drug Code	Oral Contraceptive Pill
525440631	National Drug Code	Oral Contraceptive Pill
525440847	National Drug Code	Oral Contraceptive Pill
525440848	National Drug Code	Oral Contraceptive Pill
525440892	National Drug Code	Oral Contraceptive Pill
525440936	National Drug Code	Oral Contraceptive Pill
525440940	National Drug Code	Oral Contraceptive Pill
525440949	National Drug Code	Oral Contraceptive Pill
525440950	National Drug Code	Oral Contraceptive Pill
525440951	National Drug Code	Oral Contraceptive Pill
525440953	National Drug Code	Oral Contraceptive Pill
525440954	National Drug Code	Oral Contraceptive Pill
525440959	National Drug Code	Oral Contraceptive Pill
525440966	National Drug Code	Oral Contraceptive Pill
525440967	National Drug Code	Oral Contraceptive Pill
525440981	National Drug Code	Oral Contraceptive Pill
525440982	National Drug Code	Oral Contraceptive Pill
532170091	National Drug Code	Oral Contraceptive Pill
545690689	National Drug Code	Oral Contraceptive Pill
545695493	National Drug Code	Oral Contraceptive Pill
545695796	National Drug Code	Oral Contraceptive Pill
545695816	National Drug Code	Oral Contraceptive Pill
548684369	National Drug Code	Oral Contraceptive Pill
548684730	National Drug Code	Oral Contraceptive Pill
548684744	National Drug Code	Oral Contraceptive Pill
548684754	National Drug Code	Oral Contraceptive Pill
548684814	National Drug Code	Oral Contraceptive Pill
548684828	National Drug Code	Oral Contraceptive Pill

© 2023 Bodas M et al. *JAMA Health Forum*.

548685326	National Drug Code	Oral Contraceptive Pill
548685826	National Drug Code	Oral Contraceptive Pill
548686210	National Drug Code	Oral Contraceptive Pill
552890887	National Drug Code	Oral Contraceptive Pill
580164827	National Drug Code	Oral Contraceptive Pill
651620316	National Drug Code	Oral Contraceptive Pill
651620347	National Drug Code	Oral Contraceptive Pill
669930611	National Drug Code	Oral Contraceptive Pill
669930615	National Drug Code	Oral Contraceptive Pill
681800837	National Drug Code	Oral Contraceptive Pill
681800838	National Drug Code	Oral Contraceptive Pill
681800843	National Drug Code	Oral Contraceptive Pill
681800844	National Drug Code	Oral Contraceptive Pill
681800846	National Drug Code	Oral Contraceptive Pill
681800848	National Drug Code	Oral Contraceptive Pill
681800854	National Drug Code	Oral Contraceptive Pill
681800857	National Drug Code	Oral Contraceptive Pill
681800864	National Drug Code	Oral Contraceptive Pill
681800865	National Drug Code	Oral Contraceptive Pill
681800866	National Drug Code	Oral Contraceptive Pill
681800873	National Drug Code	Oral Contraceptive Pill
681800875	National Drug Code	Oral Contraceptive Pill
681800876	National Drug Code	Oral Contraceptive Pill
681800877	National Drug Code	Oral Contraceptive Pill
681800880	National Drug Code	Oral Contraceptive Pill
681800882	National Drug Code	Oral Contraceptive Pill
681800886	National Drug Code	Oral Contraceptive Pill
681800892	National Drug Code	Oral Contraceptive Pill
681800893	National Drug Code	Oral Contraceptive Pill
681800898	National Drug Code	Oral Contraceptive Pill
681800899	National Drug Code	Oral Contraceptive Pill
681800902	National Drug Code	Oral Contraceptive Pill
681800903	National Drug Code	Oral Contraceptive Pill
684620132	National Drug Code	Oral Contraceptive Pill
684620303	National Drug Code	Oral Contraceptive Pill
684620305	National Drug Code	Oral Contraceptive Pill
684620309	National Drug Code	Oral Contraceptive Pill
684620316	National Drug Code	Oral Contraceptive Pill
684620318	National Drug Code	Oral Contraceptive Pill
684620388	National Drug Code	Oral Contraceptive Pill
684620394	National Drug Code	Oral Contraceptive Pill
684620556	National Drug Code	Oral Contraceptive Pill
684620565	National Drug Code	Oral Contraceptive Pill

© 2023 Bodas M et al. *JAMA Health Forum*.

684620637	National Drug Code	Oral Contraceptive Pill
684620646	National Drug Code	Oral Contraceptive Pill
684620656	National Drug Code	Oral Contraceptive Pill
684620657	National Drug Code	Oral Contraceptive Pill
684620672	National Drug Code	Oral Contraceptive Pill
684620719	National Drug Code	Oral Contraceptive Pill
684620720	National Drug Code	Oral Contraceptive Pill
684620733	National Drug Code	Oral Contraceptive Pill
692381531	National Drug Code	Oral Contraceptive Pill
758540601	National Drug Code	Oral Contraceptive Pill
763880283	National Drug Code	Oral Contraceptive Pill
000520273	National Drug Code	Birth Control Ring
353560410	National Drug Code	Birth Control Ring
548684832	National Drug Code	Birth Control Ring

© 2023 Bodas M et al. *JAMA Health Forum*.

eTable 2. Demographic Characteristics of Physicians in the Sample That Provided IUDs and Implants

	1	2	3	4	5	6	7	8	9	10
	All physicians in the sample	Physicians that provided at least one IUD or implant	Family Medicine physicians	Family Medicine physicians that provided at least one IUD or implant	Internal Medicine physicians	Internal Medicine physicians that provided at least one IUD or implant	OBGYN	OBGYN physicians that provided at least one IUD or implant	Pediatricians	Pediatricians that provided at least one IUD or implant
Physicians (N)	251017	25115	86106	7262	85095	606	35994	16481	43822	766
Practice Characteristics, mean (SD)										
Mean Number of Beneficiaries Provided IUDs and Implants	1.6 (19.7)	15.6 (60.5)	0.8 (18.6)	9.8 (63.5)	0.1 (4.9)	7.7 (57.6)	8.7 (41.9)	19.0 (60.3)	0.1 (2.4)	6.1 (17.3)
Mean Number of Female Reproductive Age Medicaid Beneficiaries	97.6 (229.4)	357.8 (521.9)	101.1 (213.3)	276.1 (519.5)	51.1 (133.6)	548.2 (830.2)	249.3 (415.1)	387.7 (507.3)	56.4 (105.4)	337.4 (423.1)
Specialty, No. (%)										
Family Medicine	86106 (34)	7262 (29)								
Internal Medicine	85095 (34)	606 (2)								
OB-GYN	35994 (14)	16481 (66)								
Pediatrics	43822 (17)	766 (3)								
Physician Characteristics, No. (%)										
Male,	134643 (54)	10640 (42)	51363 (60)	2981 (41)	52185 (61)	395 (65)	14967 (42)	7003 (42)	16128 (37)	261 (34)
Female	116374 (46)	14475 (58)	34743 (40)	4281 (59)	32910 (39)	211 (35)	21027 (58)	9478 (58)	27694 (63)	505 (66)
Doctors of Osteopathy	22886 (9)	2130 (8)	13689 (16)	834 (11)	4792 (6)	32 (5)	2364 (7)	1237 (8)	2041 (5)	27 (4)
International medical graduates	70589 (28)	3446 (14)	20448 (24)	1005 (14)	33935 (40)	167 (28)	5190 (14)	2162 (13)	11016 (25)	112 (15)
Age, mean (SD), Years	49.2 (12.6)	47.7 (11.0)	49.8 (12.6)	45.0 (10.7)	48.7 (12.4)	50.6 (10.5)	48.7 (12.7)	48.7 (10.9)	49.4 (12.6)	48.7 (11.2)
Age 25 to 34 Years	36554 (15)	3052 (12)	11678 (14)	1349 (19)	12924 (15)	43 (7)	5835 (16)	1588 (10)	6117 (14)	72 (9)
Age 35 to 44 Years	60446 (24)	7771 (31)	20209 (23)	2530 (35)	20995 (25)	137 (23)	8730 (24)	4878 (30)	10512 (24)	226 (30)
Age 45 to 54 Years	65942 (26)	7159 (29)	22125 (26)	1800 (25)	22927 (27)	197 (33)	9238 (26)	4937 (30)	11652 (27)	225 (29)
Age 55 to 64 Years	56435 (22)	5224 (21)	20542 (24)	1237 (17)	18618 (22)	172 (28)	7658 (21)	3643 (22)	9617 (22)	172 (22)
Age More than 65 Years	31640 (13)	1909 (8)	11552 (13)	346 (5)	9631 (11)	57 (9)	4533 (13)	1435 (9)	5924 (14)	71 (9)
Physician Located in a Rural County	15463 (6)	1928 (8)	9388 (11)	1041 (14)	3365 (4)	58 (10)	1370 (4)	790 (5)	1340 (3)	39 (5)
County Characteristics, mean (SD)										
Mean county female population 15 to 44 years	278929.4 (446138.1)	184673.2 (296620.1)	233379.1 (427807.6)	158540.8 (287621.2)	304965.9 (459168.3)	152448.5 (285224.3)	304593.8 (451487.9)	199908.5 (302256.9)	296793.0 (444326.6)	130114.0 (235513.4)

© 2023 Bodas M et al. *JAMA Health Forum*.

County % population below poverty line	14.4 (5.1)	14.4 (4.9)	14.5 (5.0)	14.0 (4.6)	14.5 (5.1)	16.4 (4.6)	14.5 (5.1)	14.4 (5.1)	14.2 (5.2)	15.2 (4.6)
County % population AIAN	1.6 (3.7)	1.6 (3.4)	1.8 (4.5)	2.0 (4.0)	1.5 (3.2)	1.0 (3.3)	1.5 (3.1)	1.4 (3.1)	1.5 (3.4)	1.2 (2.6)
County % population Asian	6.6 (7.2)	5.2 (6.1)	5.4 (6.4)	5.0 (6.1)	7.3 (7.7)	3.5 (4.8)	7.1 (7.5)	5.4 (6.2)	7.2 (7.4)	3.9 (5.1)
County % population Black	14.8 (14.0)	14.2 (14.3)	12.2 (13.0)	9.7 (12.2)	16.1 (14.3)	19.8 (19.1)	16.4 (14.3)	15.9 (14.4)	16.0 (14.1)	17.5 (16.3)
County % population Hispanic	18.0 (16.2)	15.1 (14.8)	16.9 (16.6)	14.3 (13.9)	18.2 (15.6)	10.2 (11.8)	18.9 (16.3)	15.9 (15.4)	19.1 (16.5)	10.9 (11.3)
County % population White	74.2 (15.5)	76.3 (15.8)	77.8 (14.8)	80.4 (14.3)	72.3 (15.6)	73.5 (19.5)	72.3 (15.4)	74.6 (15.9)	72.5 (15.3)	75.0 (16.8)
State Policy Characteristics, No. (%)										
Practice in a state that expanded Medicaid 2016,	174529 (70)	15697 (63)	56915 (66)	4926 (68)	62772 (74)	155 (26)	24609 (68)	10365 (63)	30233 (69)	251 (33)
Practice in a state with Medicaid family planning waiver,	165096 (66)	15606 (62)	56487 (66)	4617 (64)	56304 (66)	161 (27)	23611 (66)	10537 (64)	28694 (65)	291 (38)

NOTES - Reproductive age = 15 to 44 years
County - county in which a physician is located
AIAN - American Indian/Alaska Native
OBGYN - obstetrics and gynecology
SD - standard deviation
SOURCES - Transformed Medicaid Statistical Information System (T-MSIS) data, 2016; American Medical Association (AMA) Masterfile (2016); American Community Survey (ACS) and Kaiser Family Foundation

© 2023 Bodas M et al. *JAMA Health Forum*.

**eTable 3.** Demographic Characteristics of Physicians in the Sample Prescribing at Least 1 Pill, Patch, or Ring										
	1	2	3	4	7	8	5	6	9	10
	All physicians in the sample	Physicians that prescribed at least one pill patch or ring	Family Medicine physicians	Family Medicine physicians that prescribed at least one pill patch or ring	Internal Medicine physicians	Internal Medicine physicians that prescribed at least one pill patch or ring	OBGYN	OBGYN physicians that prescribed at least one pill patch or ring	Pediatricians	Pediatricians that prescribed at least one pill patch or ring
Physicians (N)	251017	121167	86106	51032	85095	21400	35994	29833	43822	18902
Practice Characteristics, mean (SD)										
Mean Number of Beneficiaries Provided At Least One Pill, Patch and Ring	5.8 (22.3)	12.1 (30.9)	4.4 (18.0)	7.5 (23.0)	1.1 (5.5)	4.3 (10.4)	24.6 (46.1)	29.7 (49.2)	2.4 (7.1)	5.6 (10.0)
Mean Number of Female Reproductive Age Medicaid Beneficiaries	97.6 (229.4)	150.6 (289.2)	101.1 (213.3)	129.1 (251.6)	51.1 (133.6)	103.5 (214.9)	249.3 (415.1)	264.7 (411.6)	56.4 (105.4)	82.1 (129.0)
Specialty, No. (%)										
Family Medicine	86106 (34)	51032 (42)								
Internal Medicine	85095 (34)	21400 (18)								
OB-GYN	35994 (14)	29833 (25)								
Pediatrics	43822 (17)	18902 (16)								
Physician Characteristics, No. (%)										
Male	134643 (54)	55397 (46)	51363 (60)	26909 (53)	52185 (61)	10734 (50)	14967 (42)	12106 (41)	16128 (37)	5648 (30)
Female	116374 (46)	65770 (54)	34743 (40)	24123 (47)	32910 (39)	10666 (50)	21027 (58)	17727 (59)	27694 (63)	13254 (70)
Doctors of Osteopathy	22886 (9)	12000 (10)	13689 (16)	7944 (16)	4792 (6)	1120 (5)	2364 (7)	2038 (7)	2041 (5)	898 (5)
International medical graduates	70589 (28)	28247 (23)	20448 (24)	11617 (23)	33935 (40)	8030 (38)	5190 (14)	4295 (14)	11016 (25)	4305 (23)
Age, mean (SD), Years	49.2 (12.6)	48.5 (12.1)	49.8 (12.6)	48.1 (12.2)	48.7 (12.4)	49.6 (11.4)	48.7 (12.7)	48.4 (12.4)	49.4 (12.6)	48.5 (11.8)
Age 25 to 34 Years	36554 (15)	17853 (15)	11678 (14)	8159 (16)	12924 (15)	2425 (11)	5835 (16)	4808 (16)	6117 (14)	2461 (13)
Age 35 to 44 Years	60446 (24)	30509 (25)	20209 (23)	13189 (26)	20995 (25)	4877 (23)	8730 (24)	7350 (25)	10512 (24)	5093 (27)
Age 45 to 54 Years	65942 (26)	33212 (27)	22125 (26)	13293 (26)	22927 (27)	6677 (31)	9238 (26)	7877 (26)	11652 (27)	5365 (28)
Age 55 to 64 Years	56435 (22)	27056 (22)	20542 (24)	11277 (22)	18618 (22)	5338 (25)	7658 (21)	6374 (21)	9617 (22)	4067 (22)
Age More than 65 Years	31640 (13)	12537 (10)	11552 (13)	5114 (10)	9631 (11)	2083 (10)	4533 (13)	3424 (11)	5924 (14)	1916 (10)
Physician Located in a Rural County	15463 (6)	8393 (7)	9388 (11)	5597 (11)	3365 (4)	908 (4)	1370 (4)	1131 (4)	1340 (3)	757 (4)
County Characteristics, mean (SD)										
Mean county female population 15 to 44 years	278929.4 (446138.1)	286992.4 (470841.8)	233379.1 (427807.6)	247221.6 (454559.9)	304965.9 (459168.3)	369812.6 (537280.3)	304593.8 (451487.9)	300969.8 (455095.5)	296793.0 (444326.6)	278540.4 (444041.6)

© 2023 Bodas M et al. *JAMA Health Forum*.

County % population below poverty line	14.4 (5.1)	14.5 (5.0)	14.5 (5.0)	14.4 (4.9)	14.5 (5.1)	14.6 (5.1)	14.5 (5.1)	14.5 (5.0)	14.2 (5.2)	14.3 (5.1)
County % population AIAN	1.6 (3.7)	1.6 (3.5)	1.8 (4.5)	1.8 (4.0)	1.5 (3.2)	1.5 (3.2)	1.5 (3.1)	1.5 (3.0)	1.5 (3.4)	1.5 (3.1)
County % population Asian	6.6 (7.2)	6.5 (7.2)	5.4 (6.4)	5.6 (6.6)	7.3 (7.7)	8.1 (7.9)	7.1 (7.5)	7.0 (7.4)	7.2 (7.4)	6.8 (7.1)
County % population Black	14.8 (14.0)	13.9 (13.6)	12.2 (13.0)	11.5 (12.5)	16.1 (14.3)	15.3 (13.7)	16.4 (14.3)	16.2 (14.3)	16.0 (14.1)	14.9 (13.9)
County % population Hispanic	18.0 (16.2)	18.1 (16.3)	16.9 (16.6)	17.0 (16.4)	18.2 (15.6)	19.4 (15.9)	18.9 (16.3)	18.8 (16.4)	19.1 (16.5)	18.3 (16.4)
County % population White	74.2 (15.5)	75.1 (15.2)	77.8 (14.8)	78.3 (14.5)	72.3 (15.6)	72.2 (15.2)	72.3 (15.4)	72.5 (15.5)	72.5 (15.3)	74.0 (15.2)
State Policy Characteristics, No. (%)										
Practice in a state that expanded Medicaid 2016,	174529 (70)	87262 (72)	56915 (66)	35933 (70)	62772 (74)	17506 (82)	24609 (68)	20541 (69)	30233 (69)	13282 (70)
Practice in a state with Medicaid family planning waiver,	165096 (66)	80319 (66)	56487 (66)	34061 (67)	56304 (66)	14002 (65)	23611 (66)	19639 (66)	28694 (65)	12617 (67)

NOTES - Reproductive age = 15 to 44 years
County - county in which a physician is located
AIAN - American Indian/Alaska Native
OBGYN - obstetrics and gynecology
SD - standard deviation
SOURCES - Transformed Medicaid Statistical Information System (T-MSIS) data, 2016; American Medical Association (AMA) Masterfile (2016); American Community Survey (ACS) and Kaiser Family Foundation

© 2023 Bodas M et al. *JAMA Health Forum*.

eTable 4. Multivariate Regression Results for the Full Analytical Sample (N = 251 017)								
Outcome	1<br>Total Number of Medicaid Beneficiaries Provided IUDs and Implants in 2016		2<br>Total Number of Medicaid Beneficiaries Prescribed Pill Patch and Ring in 2016		3<br>Odds of Providing IUDs and Implants to Medicaid Beneficiaries by Physicians 2016		4<br>Odds of Prescribing Pill Patch and Ring in 2016 to Medicaid Beneficiaries by Physicians 2016	
	Average Marginal Effect	95% Confidence Interval	Average Marginal Effect	95% Confidence Interval	Odds Ratio	95% Confidence Interval	Odds Ratio	95% Confidence Interval
Physician Characteristics								
Female	0.51**	(0.26 - 0.76)	2.39**	(1.91 - 2.87)	1.62**	(1.48 - 1.77)	1.88**	(1.76 - 2.00)
Doctor of Osteopathy	-0.50**	(-0.67 - -0.34)	-0.20	(-0.67 - 0.28)	0.68**	(0.61 - 0.76)	1.03	(0.95 - 1.10)
International Medical Graduate	-1.13**	(-1.29 - -0.97)	-1.58**	(-2.38 - -0.79)	0.39**	(0.33 - 0.46)	0.64**	(0.58 - 0.71)
Age 35 to 44 Years	1.33**	(1.02 - 1.64)	3.46**	(2.54 - 4.39)	1.69**	(1.53 - 1.88)	1.10	(0.99 - 1.22)
Age 45 to 54 Years	1.03**	(0.72 - 1.35)	4.03**	(2.86 - 5.19)	1.39**	(1.19 - 1.61)	1.15*	(1.01 - 1.29)
Age 55 to 64 Years	0.67**	(0.41 - 0.93)	3.97**	(2.84 - 5.11)	1.20*	(1.01 - 1.43)	1.11	(0.99 - 1.24)
Age More than 65 Years	0.38	(-0.40 - 1.17)	4.06**	(2.35 - 5.77)	0.89	(0.73 - 1.08)	0.89	(0.78 - 1.01)
County Characteristics								
Rural	-0.68**	(-0.92 - -0.43)	-0.67	(-1.35 - 0.00)	0.84*	(0.71 - 0.98)	1.18*	(1.03 - 1.36)
Log of county female population 15 to 44 years	-0.28**	(-0.46 - -0.09)	-0.16	(-0.50 - 0.19)	0.93	(0.86 - 1.01)	0.99	(0.94 - 1.05)
County percent population below poverty line	0.04**	(0.01 - 0.08)	0.24**	(0.15 - 0.33)	1.01	(0.99 - 1.03)	1.03**	(1.01 - 1.04)
County percent population Asian	-0.02	(-0.04 - 0.00)	-0.12**	(-0.19 - -0.06)	0.98*	(0.96 - 1.00)	1.00	(0.99 - 1.01)
County percent population Black	0.02	(-0.01 - 0.05)	-0.01	(-0.08 - 0.06)	0.99	(0.99 - 1.00)	0.99**	(0.98 - 0.99)
County percent population Hispanic	-0.01	(-0.02 - 0.00)	-0.02	(-0.05 - 0.01)	0.99	(0.98 - 1.00)	1.00	(0.99 - 1.01)
County percent population AIAN	0.02**	(0.01 - 0.03)	0.02	(-0.02 - 0.07)	1.00	(0.98 - 1.01)	0.99*	(0.98 - 1.00)
State Policy Characteristics								
State expanded Medicaid by 2016	-0.06	(-0.45 - 0.34)	2.52**	(1.24 - 3.80)	0.85	(0.64 - 1.14)	1.27	(0.99 - 1.63)
State has a family planning waiver	0.26	(-0.18 - 0.70)	0.38	(-0.88 - 1.65)	0.86	(0.58 - 1.27)	1.05	(0.82 - 1.34)

Confidence intervals in parentheses
** p<0.01, * p<0.05
AIAN - American Indian/Alaska Native
OBGYN – obstetrics and gynecology

© 2023 Bodas M et al. *JAMA Health Forum*.

eFigure 2. Overview of State Selection Process

T-MSIS Data Quality Assessment and State Selection for Analysis

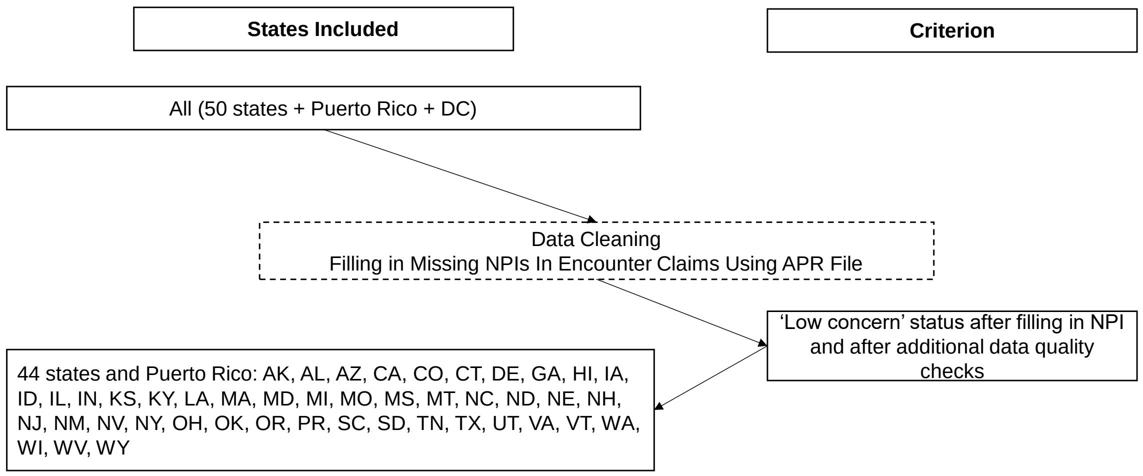

A. Initial data quality assessment

To perform an initial assessment of states' data quality, we referred to Data Quality (DQ) Atlas (see eTable 4). Since being able to identify a physician that provided a particular service is crucial for our analysis, we required states to have 'low concern' status on the topic 'Servicing Provider NPI – Other Services (OT) File' (19 states) and on the topic 'Prescribing Provider NPI – Pharmacy (RX) File' (44 states).

However, only **16 states** met our selection criterion and relaxing it to include states with 'medium concern' data quality would have added only 13 more states. Therefore, to expand our sample size, we performed additional data cleaning based on information in the T-MSIS Annual Provider (APR) File.

B. Data cleaning

I. Understanding T-MSIS file structure

Following is a brief summary of the contents of files in T-MSIS 2016.

a) Other Services (OT) file: This file includes encounter data. It includes:
   a. records for physician services, outpatient hospital institutional utilization, lab/X-ray, clinic services etc.
   b. diagnosis and procedure codes for an encounter
   c. billing and servicing provider National Provider Identifiers (NPIs)
   d. billing and servicing state Provider Identification Numbers: These are state-assigned unique numbers to identify the provider who billed or provided a particular service

b) Pharmacy (RX) file: This file includes information about prescriptions filled for Medicaid beneficiaries. It includes:
   a. National Drug Codes (NDCs)
   b. billing provider NPIs
   c. billing state Provider Identification Numbers

c) Annual Provider (APR) file: This file has information about providers of Medicaid services that can be linked to individual service record. It includes:
   a. provider NPIs
   b. provider state Provider Identification Numbers

II. Linking NPIs with State Provider Numbers

Since each physician has a unique NPI and a unique Provider Identification Number (in each state), it was possible to create a linkage between these two data elements.

© 2023 Bodas M et al. *JAMA Health Forum*.

III. Filling in missing NPIs in encounter and prescription data

Next, in cases where the servicing provider NPI was missing for a particular claim but the servicing state Provider Identification Number was present, we used the ‘NPI-State Provider Identification Number’ linkage and filled in the servicing providing NPI for that particular claim (see figure below).

© 2023 Bodas M et al. *JAMA Health Forum*.

**eTable 5.** T-MSIS DQ Atlas Single Topic Summary			
**State**	**Servicing Provider NPI - OT**	**Prescribing Provider NPI - RX**	**Meets Criterion**
Alabama	Low concern	Low concern	Yes
Alaska	Medium concern	Low concern	No
Arizona	Low concern	Low concern	Yes
Arkansas	**Unusable**	Low concern	No
California	Medium concern	Low concern	No
Colorado	Medium concern	Low concern	No
Connecticut	**High concern**	Low concern	No
Delaware	Medium concern	Low concern	No
District of Columbia	Medium concern	**Unusable**	No
Florida	**High concern**	**Unusable**	No
Georgia	Low concern	Low concern	Yes
Hawaii	Low concern	Low concern	Yes
Idaho	Medium concern	Low concern	No
Illinois	**High concern**	Low concern	No
Indiana	Low concern	Low concern	Yes
Iowa	Low concern	Low concern	Yes
Kansas	**High concern**	Low concern	No
Kentucky	Low concern	Low concern	Yes
Louisiana	Low concern	Low concern	Yes
Maine	Low concern	**Unusable**	No
Maryland	Medium concern	Low concern	No
Massachusetts	**High concern**	Low concern	No
Michigan	Low concern	Low concern	Yes
Minnesota	Low concern	Low concern	Yes
Mississippi	Low concern	**Unusable**	No
Missouri	**High concern**	Low concern	No
Montana	**High concern**	**High concern**	No
Nebraska	Medium concern	Low concern	No
Nevada	Low concern	Low concern	Yes
New Hampshire	Medium concern	Low concern	No
New Jersey	Low concern	Low concern	Yes
New Mexico	Low concern	Low concern	Yes
New York	Medium concern	Low concern	No
North Carolina	**High concern**	Low concern	No
North Dakota	**High concern**	Low concern	No
Ohio	Medium concern	Medium concern	No
Oklahoma	**High concern**	Low concern	No
Oregon	Low concern	Low concern	Yes
Pennsylvania	**Unusable**	Low concern	No
Puerto Rico	Medium concern	Low concern	No

© 2023 Bodas M et al. *JAMA Health Forum*.

Rhode Island	**High concern**	**Unusable**	No
South Carolina	**High concern**	Low concern	No
South Dakota	Low concern	**High concern**	No
Tennessee	**High concern**	Low concern	No
Texas	**High concern**	Low concern	No
Utah	**Medium concern**	Low concern	No
Vermont	**Medium concern**	Low concern	No
Virginia	Low concern	Low concern	Yes
Washington	**High concern**	Low concern	No
West Virginia	**Medium concern**	Low concern	No
Wisconsin	**Medium concern**	Low concern	No
Wyoming	Low concern	Low concern	Yes

SOURCE: 2016 Transformed Medicaid Statistical Information System Analytic Files from and the Centers for Medicare and Medicaid Services Data Quality Atlas assessments for TMSIS Analytic Files (TAF) data year 2016, available at https://www.medicaid.gov/dq-atlas/landing/topics
NOTES: “Low Concern” and “Medium Concern” determinations come from the Centers for Medicare and Medicaid Services Data Quality Atlas assessments for TMSIS Analytic Files (TAF) data year 2016

© 2023 Bodas M et al. *JAMA Health Forum*.

eFigure 3. An Example of Filling in NPIs in T-MSIS Encounter Claims

Before filling in NPIs					
**Claim ID**	**Beneficiary ID**	**Procedure Code**	**Diagnosis Code**	**Servicing Provider NPI**	**Servicing Provider Identification Number**
CLM_ID_001	BENE_ID_001	58300	Z30.9	1234567890 ◄	► STATE_A_001
CLM_ID_002	BENE_ID_002	58300	Z30.9	missing	STATE_A_001
CLM_ID_003	BENE_ID_003	58300	Z30.9	missing	STATE_A_001

↓

After filling in NPIs					
**Claim ID**	**Beneficiary ID**	**Procedure Code**	**Diagnosis Code**	**Servicing Provider NPI**	**Servicing Provider Identification Number**
CLM_ID_001	BENE_ID_001	58300	Z30.9	1234567890 ◄	► STATE_A_001
CLM_ID_002	BENE_ID_002	58300	Z30.9	1234567890 ◄	► STATE_A_001
CLM_ID_003	BENE_ID_003	58300	Z30.9	1234567890 ◄	► STATE_A_001

This enabled us to improve the quality of many states' encounter data on servicing and prescribing provider NPIs.

We would like to note that state categorization based on missingness of state-assigned Provider Identification Numbers is currently not available in the DQ Atlas.

IV. Reassessing states' data quality

As the final step of state selection for analysis, we performed additional checks on states' data.

First, we calculated the percentage of each state's total submitted claim lines that had missing servicing providing NPIs, after performing the data cleaning described earlier. We categorized states' OT file data as having 'low concern' if less than 10% of their claim lines had missing servicing NPIs. Similarly, states which had missing servicing provider NPIs in 10 to 20% of the claim lines were categorized as having 'medium concern' data. States which did not have servicing NPIs in 20 to 50% of their claim lines were categorized as having 'high concern' data and if more than 50% of claim lines had missing servicing provider NPIs, data from such states was categorized as 'unusable'. The categorization used for RX files was similar (0-10% claim lines missing prescribing NPIs = low concern; 10-20% missing = medium concern; 20-50% missing = high concern; more than 50% missing = unusable)

We excluded states that had 'high concern' or 'unusable' data quality status for either file. The result of this recategorization of states is summarized in eTable 5.

During this process, we also checked for unusual data patterns and for data anomalies. We found that Minnesota's T-MSIS 2016 OT file reported NPIs and State Provider Numbers of managed care organizations and not of individual providers in the 'Servicing Provider NPI' and 'Servicing Provider Identification Number' fields. Since this is inaccurate, we categorized Minnesota's data as 'unusable' for analysis. On the other hand, Mississippi's RX data had received 'unusable' status in the DQ Atlas since the 'Prescribing Provider NPI' filed in the state's OT file had a lot of missing data. After data cleaning, we were able to remedy this situation and we subsequently categorized Mississippi's data as 'low concern'.

After performing data quality reassessment, our final analytical sample included the 44 states and Puerto Rico. We excluded 6 states (Arkansas, Florida, Maine, Minnesota, Pennsylvania, Rhode Island) and Washington, DC from the analysis.

© 2023 Bodas M et al. *JAMA Health Forum*.

**eTable 6.** State Data Quality Assessment and Sample Selection			
**State**	**Post-cleaning Servicing Provider NPI – OT**	**Post-cleaning Prescribing Provider NPI – RX**	**Sample Inclusion**
Alabama	Low Concern	Low Concern	Included
Alaska	Low Concern	Low Concern	Included
Arizona	Low Concern	Low Concern	Included
Arkansas	**Unusable**	Low Concern	Excluded
California	**Medium Concern**	Low Concern	Included
Colorado	**Medium Concern**	Low Concern	Included
Connecticut	Low Concern	Low Concern	Included
Delaware	Low Concern	Low Concern	Included
District Of Columbia	**Medium Concern**	**Unusable**	Excluded
Florida	Low Concern	**Unusable**	Excluded
Georgia	Low Concern	Low Concern	Included
Hawaii	Low Concern	Low Concern	Included
Idaho	Low Concern	Low Concern	Included
Illinois	Low Concern	Low Concern	Included
Indiana	Low Concern	Low Concern	Included
Iowa	Low Concern	Low Concern	Included
Kansas	Low Concern	Low Concern	Included
Kentucky	Low Concern	Low Concern	Included
Louisiana	Low Concern	Low Concern	Included
Maine	Low Concern	**Unusable**	Excluded
Maryland	Low Concern	Low Concern	Included
Massachusetts	Low Concern	Low Concern	Included
Michigan	Low Concern	Low Concern	Included
Minnesota	**Unusable**	Low Concern	Excluded
Mississippi	Low Concern	Low Concern	Included
Missouri	Low Concern	Low Concern	Included
Montana	Low Concern	Low Concern	Included
Nebraska	Low Concern	Low Concern	Included
Nevada	Low Concern	Low Concern	Included
New Hampshire	Low Concern	Low Concern	Included
New Jersey	Low Concern	Low Concern	Included
New Mexico	Low Concern	Low Concern	Included
New York	Low Concern	Low Concern	Included
North Carolina	Low Concern	Low Concern	Included
North Dakota	Low Concern	Low Concern	Included
Ohio	Low Concern	Low Concern	Included
Oklahoma	Low Concern	Low Concern	Included
Oregon	Low Concern	Low Concern	Included

© 2023 Bodas M et al. *JAMA Health Forum*.

Pennsylvania	**High Concern**	Low Concern	Excluded
Puerto Rico	Low Concern	Low Concern	Included
Rhode Island	Low Concern	**Unusable**	Excluded
South Carolina	Low Concern	Low Concern	Included
South Dakota	Low Concern	**Medium Concern**	Included
Tennessee	Low Concern	Low Concern	Included
Texas	Low Concern	Low Concern	Included
Utah	**Medium Concern**	Low Concern	Included
Vermont	Low Concern	Low Concern	Included
Virginia	Low Concern	Low Concern	Included
Washington	Low Concern	Low Concern	Included
West Virginia	**Medium Concern**	Low Concern	Included
Wisconsin	Low Concern	Low Concern	Included
Wyoming	Low Concern	Low Concern	Included

© 2023 Bodas M et al. *JAMA Health Forum*.

eFigure 4. Physician State Reassignment Process Example

We assigned a state to each physician in the sample based on the number of Medicaid claims submitted. For example, if a physician was located in state A according to the NPPES but had most or all of their claims submitted to state B Medicaid program, then for the purpose of this analysis, we assigned state B as that physician's state (see Physicians #3 and #4 in the example below).

All Physicians are located in State A, according to the NPPES
Each arrow represents a claim submitted to a Medicaid program
Each physician submitted 5 claims

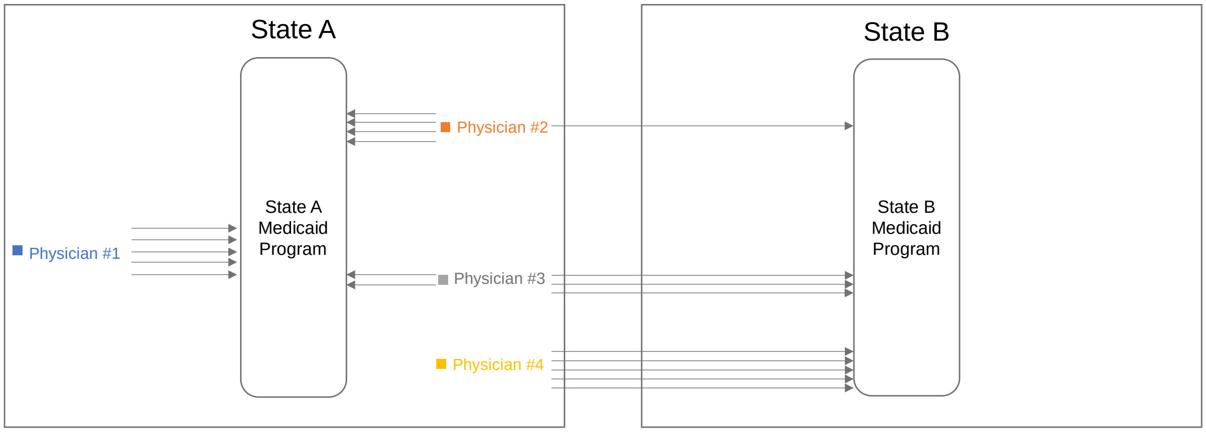

■ Assigned to the same state as noted in the NPPES and submitted claims to a single state's Medicaid program (state A)

■ Assigned to the same state as noted in the NPPES and submitted claims to multiple states' Medicaid programs (state A and state B)

■ Assigned to a state different from their NPPES state and submitted claims to multiple states' Medicaid programs (state A and state B)

■ Assigned to a state different from their NPPES state and submitted claims to a single state's Medicaid program (state B)

© 2023 Bodas M et al. *JAMA Health Forum*.

eFigure 5. Physician State Reassignment Details

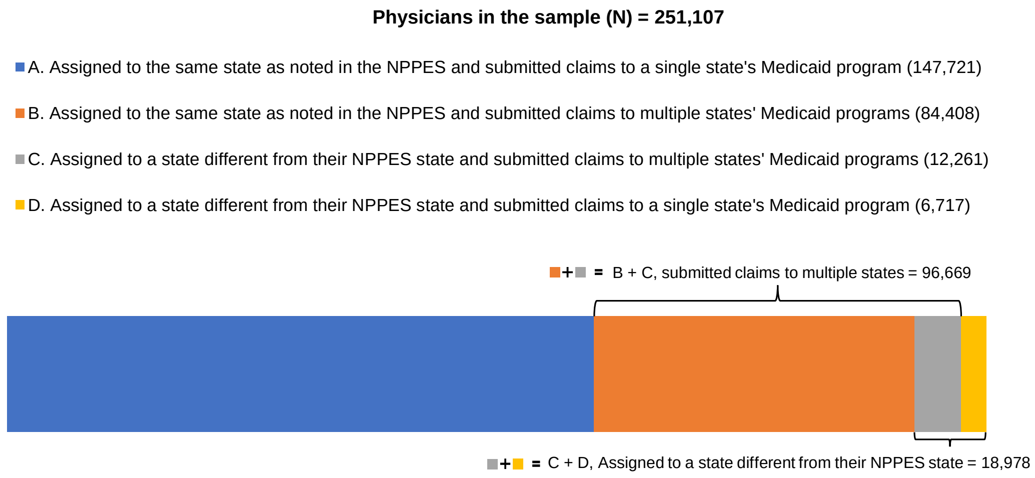

© 2023 Bodas M et al. *JAMA Health Forum*.
