# Supplementary material for: Association of Primary Care Physicians’ Individual- and Community-Level Characteristics With Contraceptive Service Provision to Medicaid Beneficiaries
Source: JAMA Health Forum. 2023 Mar 17;4(3):e230106. doi: 10.1001/jamahealthforum.2023.0106 (PMC10024198; doi:10.1001/jamahealthforum.2023.0106)
Supplement: Supplement 2. — Data Sharing Statement [file jamahealthforum-e230106-s002.pdf]

Data Sharing Statement

Bodas. Association of Primary Care Physicians' Individual- and Community-Level Characteristics With Contraceptive Service Provision to Medicaid Beneficiaries. *JAMA Health Forum*. Published March 17, 2023. doi:10.1001/jamahealthforum.2023.0106

Data
Data available: No
